# Shaping the Brain: The Emergence of Cortical Structure and Folding

**DOI:** 10.1016/j.devcel.2023.11.004

**Published:** 2023-12-18

**Authors:** Shyam K. Akula, David Exposito-Alonso, Christopher A. Walsh

**Affiliations:** 1 Division of Genetics and Genomics, Boston Children’s Hospital, Boston, MA, USA; 2 Departments of Pediatrics and Neurology, Harvard Medical School, Boston, MA, USA; 3 Allen Discovery Center for Human Brain Evolution, Boston Children’s Hospital and Harvard Medical School, Boston, MA, USA; 4 Howard Hughes Medical Institute

## Abstract

The cerebral cortex—the brain’s covering and largest region—has increased in size and complexity in humans and supports higher cognitive functions such as language and abstract thinking. There is a growing understanding of the human cerebral cortex, including the diversity and number of cell types that it contains, as well as of the developmental mechanisms that shape cortical structure and organization. In this Review, we discuss recent progress in our understanding of molecular and cellular processes, as well as mechanical forces, that regulate the folding of the cerebral cortex. Advances in human genetics, coupled with experimental modeling in gyrencephalic species, have provided insights into the central role of cortical progenitors in the gyrification and evolutionary expansion of the cerebral cortex. These studies are essential for understanding the emergence of structural and functional organization during cortical development and the pathogenesis of neurodevelopmental disorders associated with cortical malformations.

## eTOC

During brain development, neural stem cells generate many cell types that assemble to build the cerebral cortex, the substrate for higher cognitive functions. In a review, Akula and Exposito-Alonso et al. examine our current understanding of the mechanisms underlying cortical development and folding, and how alterations may cause brain malformations.

## INTRODUCTION

The cerebral cortex constitutes the majority of the human brain by mass, and the neocortex—the newest evolutionary addition to the cerebral cortex—has expanded extraordinarily in humans when compared to our phylogenetically closest relatives, the great apes ^1,2^. Recent progress in describing the human neocortex has revealed unique features relating to the number and diversity of cell types ^3–8^. However, our understanding of how the neocortex has acquired its shape and organization remains incomplete. Some of the remarkable human cognitive abilities that resulted from the increases in size and complexity of the neocortex are disrupted in patients with cortical malformations, neurodevelopmental disorders characterized by abnormal cortical structure and/or organization, and understanding the process of neocortical formation may give us insights into these disorders ^9–12^.

The presence of folds in the neocortex is a hallmark in many mammals, including humans. Neocortical folding is characterized by patterns of fissures called sulci and ridges called gyri, hence the name gyrencephalic for describing species with a folded cortex. The thickness of the neocortex varies across the folds: the grey matter (where neuronal cell bodies reside) is thickest at the peaks of gyri and thinnest at the deepest points of sulci. Therefore, areas corresponding to gyri and sulci can be inferred from the variation in thickness ^13,14^. Cortical folding is thought to provide compaction of the neocortical structure, allowing a larger surface area to fit in a smaller volume. In humans, primary gyri and sulci are the first convolutions to emerge during development, are the largest folds, and display consistent locations across individuals ^14^. In contrast, secondary and tertiary folds are formed later in development and vary to some degree in their locations and orientations ^15–18^. Importantly, patterns of cortical folding may have consequences for functional connectivity: for instance, neurons separated by a gyrus are more likely to be functionally connected than those separated by a sulcus ^19–21^.

Not all mammals have a gyrencephalic neocortex. Common animal models in neuroscience research such as mice and rats are lissencephalic (i.e., their cortices are smooth). Cortical gyrification was first proposed to have emerged from common lissencephalic ancestors as neocortical size increased ^22^. However, broad comparative analysis of mammalian brain anatomy suggests differently. Cortical gyrification is observed even in Monotremes and Marsupials ^23^, far removed from the common ancestor of mice and gyrencephalic species, and within Rodentia, members with larger brains such as capybaras show gyrification. Thus, in rodents, and particularly in mice and rats, lissencephaly more likely represents a derived trait, likely selected for through demands for a smaller head or less energetically expensive cortex ^24^. A gyrencephalic cortex was very likely present in the ancestral animal of all living mammals today, suggesting that cortical folding was a core feature of the early neocortex ^25,26^. In gyrencephalic species with only primary folds, such as ferrets, the sulcation pattern is essentially invariant between animals ^27^. Although mice do not develop cortical gyration, they remain useful model organisms to explore aspects of neocortical development, even related to cortical folding, because of the high degree of conservation in mechanisms of neocortical development across all mammals. Recently, understanding cortical folding has benefited from the study of cortical folding disorders in human individuals ^28^, and the more recent development of genetic manipulation in gyrencephalic models like ferrets have led to a new era of exploring the biology of cortical folding ^29–31^.

In this Review, we explore and discuss advances in our understanding of the development of cortical folding as a central process underlying the emergence of shape in the human brain. We provide a comprehensive review on the role that cortical progenitors play in the formation of cortical folding, with interdisciplinary consideration of studies of the biophysics of this process. Our attention is also focused on comparative analyses across species that have provided key insights into the cellular mechanisms underlying cortical folding. We review signaling pathways that regulate the dynamics and maintenance of cortical progenitors and concentrate on specific examples for which extensive studies of animal modeling as well as human genetics converge to inform us about their function in cortical gyrification. We discuss recent data establishing a key role for Sonic Hedgehog signaling in cortical folding, based on experiments in ferret models and genetic studies of patients with cortical malformations. Next, we highlight findings of evolutionary mechanisms that illustrate the importance of human-specific genetic changes in regulating the behavior of cortical progenitors in the developing cortex, and how these evolutionary specializations may have impacted cortical size and complexity. Finally, we provide a perspective on recent studies that point towards emerging cellular mechanisms shaping the development of neocortical gyrification.

## CORTICAL DEVELOPMENT AND NEURAL PROGENITOR CELLS

**Figure 1.**
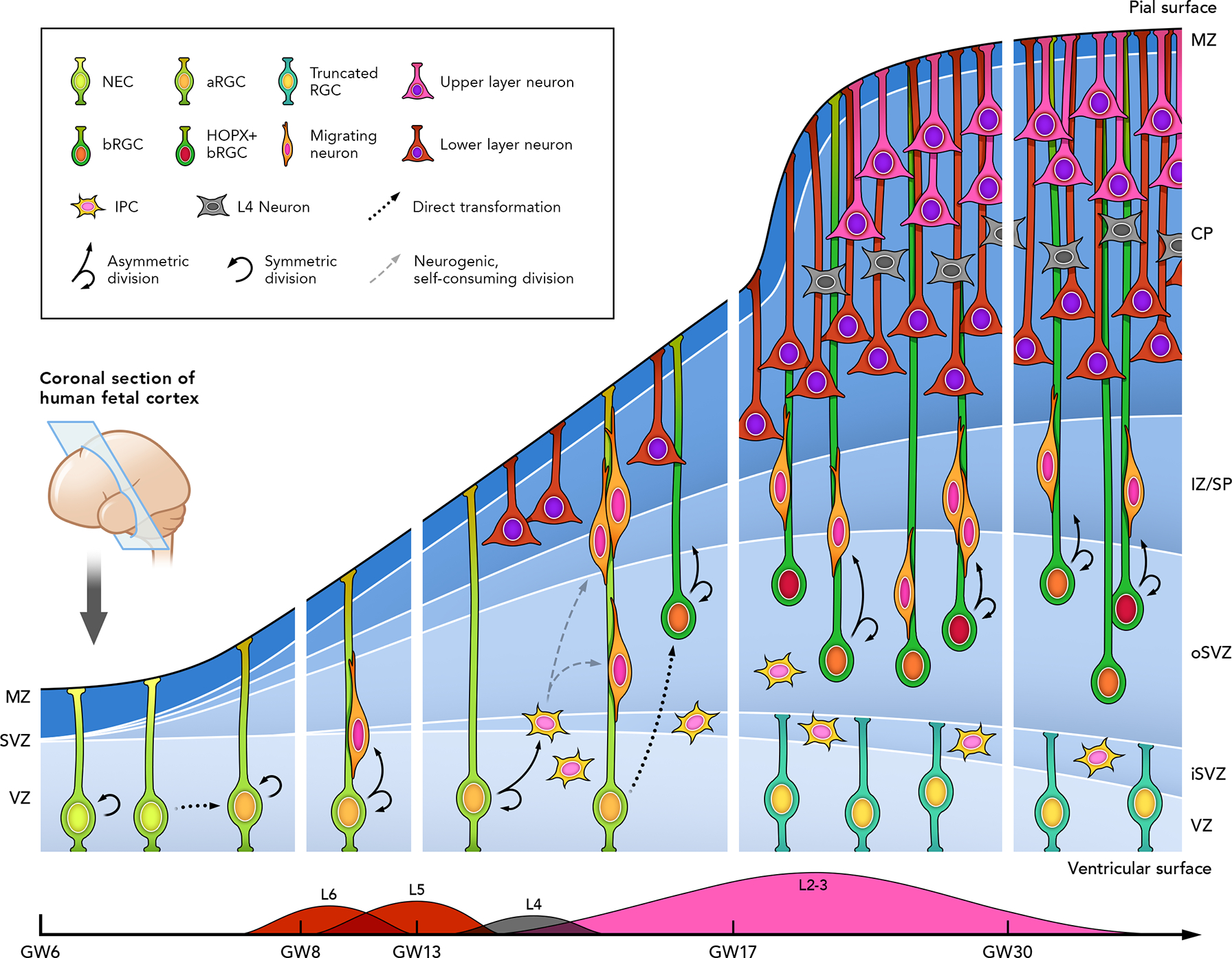
Temporal stages and progenitor types in the human developing neocortex. During early human brain development, a layer of neuroepithelial cells (NECs), spanning from the ventricular surface to the pial surface, populates the developing neural tube and undergoes self-renewing divisions to generate more NECs (symmetric divisions) in early developmental stages. They then elongate and differentiate into radial glial cells (RGCs), which also undergo symmetric divisions to expand the population of progenitors. During the neurogenic period, RGCs begin to divide asymmetrically to generate neurons while self-maintaining the progenitor pool, either generating neurons directly or producing neurons indirectly through intermediate progenitor cells (IPCs). Apical radial glial cells (aRGCs) are defined by residing in the ventricular zone (VZ) and by establishing contacts at both the apical and basal surfaces of the developing cortex. Later in development, aRGCs can also give rise to basal radial glial cells (bRGCs) by delamination of the apical belt of adherens junctions attached to the ventricular surface and translocation of their somas to the subventricular zone (SVZ). Based on marker expression, bRGCs have multiple subtypes, including HOPX+ bRGCs. Migrating neurons generated by aRGCs or bRGCs use the RG scaffold of both types of progenitors to migrate through the intermediate zone (IZ) into the developing cortical plate (CP), which contributes to the growth of the CP. By the second trimester of pregnancy (gestational week 17), aRGCs transform into truncated RGCs with their basal process terminating in the border between the inner and outer layers of the SVZ (iSVZ/oSVZ); thus, the RG scaffold becomes truncated at the iSVZ/oSVZ border. Cortical neurons are born in an inside-out fashion, with neurons destined to deeper layers (L6) born first and neurons destined to superficial layers (L2) born last. Density plots shown on the bottom represent the different neurogenic stages that preferentially generate neurons committed to each cortical layer. The extended neurogenic period for superficial layer neurons (L2–3), coincides with the expansion of bRGC proliferation and is considered a hallmark of human brain evolution.

As corticogenesis begins, neuroepithelial cells (NECs) give rise to cortical progenitor cells known as radial glial cells (RGC) that are responsible for generating diverse populations of cortical projection neurons (Figure 1). Apical RGCs (aRGCs), whose cell somata reside in the ventricular zone (VZ), initially undergo symmetric divisions to amplify the pool of progenitor cells ^32–34^. aRGCs extend long processes that span from the ventricular surface apically to the pial surface basally ^34,35^ (Figure 1). As the neurogenic period of cortical development begins, aRGCs begin also to divide asymmetrically to produce a daughter neuron and another aRGC, which is known as a neurogenic and self-renewal division ^32^. Furthermore, aRGCs can produce nonpolar, division-limited intermediate progenitors (IPCs), which upon division will generate two daughter neurons, hence amplifying the output of neurons produced during neurogenesis ^32,36–38^ (Figure 1). The vast majority of IPCs undergo their mitotic division in the subventricular zone (SVZ), although these progenitors can be found across germinal zones (VZ and SVZ), which determines their classification as apical or basal IPCs, respectively ^38–42^. bIPCs can exhibit proliferative, self-renewal capacities in primates, in contrast to mouse IPCs that generally undergo self-consuming divisions ^42^. As corticogenesis proceeds, aRGCs generate a second class of radial glial progenitors called basal RGCs (bRGCs; also known as outer radial glial cells) that reside basally within the subventricular zone (SVZ), delaminate from the apical belt of adherens junctions attached to the ventricular surface, and retain their polarity typically by extending a basal process connected to the basal surface of the developing cortex although can display remarkable diversity of morphological subtypes ^39,42–44^ (Figure 1).

The SVZ can be anatomically subdivided into an inner and an outer layer (iSVZ and oSVZ, respectively): the iSVZ contains randomly organized cells, whereas the oSVZ shows radially organized cells and is the main location of bRGCs ^42–45^. The inner fiber layer constitutes the anatomical landmark separating the iSVZ and oSVZ and emerges around embryonic day 72 in the macaque developing cortex (roughly corresponding to the beginning of the second trimester of gestation in humans) ^45^. A growing body of literature has documented that numerous successive rounds of proliferative divisions of bRGCs (in some cases, summing up to 6 division rounds) and to a lower degree of bIPCs, ensure the progressive accumulation of neurons during cortical development. These findings have formed the basis for the view that the high proliferative capacity of bRGCs may underlie the expansion of oSVZ and the increased cortical neuron numbers in primates, achieving its highest levels in humans ^42–49^.

Nascent, immature neurons in the developing cortex utilize the radial glial processes from RGCs that extend to the basal pial surface—adhering to them through cell adhesion mechanisms—to migrate radially through an intermediate zone (IZ) to finally reach the developing cortical plate (CP), where they will mature and form synaptic connections to begin elaborating developing cortical circuits (Figure 1). Thus, radial glial processes (also known as RG fibers) form cytoarchitectonic scaffolds that are essential for radial migration of neurons. The CP appears by splitting a primordial basal zone that was above the early VZ before neurogenesis, known as the pre-plate (PP), separating it into a cell-rich subplate (SP) of early pioneer neurons below the CP, and the marginal zone (MZ) above the CP; the MZ terminates at the pial surface. Although early models suggested that nascent neurons may migrate in narrow columns called “radial units” ^50^, analysis of excitatory neuron clones derived from single or few RGCs show a more conical distribution in mouse, widening as daughter neurons migrate further from the VZ. In ferrets, clonal marking and live imaging analyses of cortical slice cultures show even more prominent tangential dispersion, suggesting that migrating neurons use the radial glial scaffold as guides in general, but do not rigidly adhere to specific RG fibers, allowing them to disperse more horizontally in the developing CP of gyrencephalic species ^40,51,52^. Together with the RG fibers displaying a fanned array in sites of developing folds ^40^, these data provide key insights into the mechanisms for increased surface area in the gyrencephalic cortex (discussed in the next section).

After they are generated from the early neuroepithelium, RGCs undergo multiple rounds of symmetric division to increase the pool of progenitors and later asymmetric divisions to both generate a daughter neuron and self-renew the progenitor cell ^32,53,54^. However, as neurogenesis proceeds, individual RGCs will themselves exit the cell cycle to produce terminally differentiated daughter cells. The timing of the switch from symmetric to asymmetric (i.e., expanding the number of progenitors vs. producing neurons), as well as the timing of cell cycle exit for aRGCs and bRGCs, dictates their neurogenic potential. RGCs that proliferate for shorter times produce fewer daughter cells. Thus, regulation of the cell cycle and division mode of different types of RGCs is a determining factor for brain size and cortical thickness ^55,56^.

While the fundamental components of neurogenesis described above are conserved in all developing mammalian cortices examined ^57,58^, the relative abundance of progenitor cell types and their behaviors vary with brain size and folding pattern across species, which provides correlative implications about the cellular bases of cortical thickness, size, and gyrification ^49,56^. Gyrencephalic species like ferrets and humans have thicker and more complex SVZs relative to lissencephalic mice. Beyond increased frequency in the developing cortex, basal progenitors have greater diversity in subtype and neurogenic potential in primates than in mice ^38,49,55,56,59^. A basal progenitor type reminiscent of human bRGCs, with marked epithelial features, appears to be present only in small numbers in the SVZ of mice, which primarily rely on aRGCs and IPCs during their short neurogenic period ^47,60^. In contrast, bRGCs, rather than aRGCs, predominantly produce cortical neurons in primates, including humans, and are considered essential in the generation of superficial layer neurons, which have extraordinarily expanded in numbers and diversity in humans ^3,49,55,56,61^.

## MECHANISMS OF CORTICAL FOLDING

The process of cortical folding undergoes several developmental stages—spanning from embryonic stages through postnatal development in humans—and requires a complex interplay of mechanisms ^18,49^. On the one hand, physical studies of cortical folding suggest that mechanical forces from differential tangential expansion of the CP relative to the germinal layers (VZ and SVZ) are sufficient as an important factor to induce cortical gyration ^62^. On the other hand, twin studies indicate that gyration patterns are heritable, with patterns of sulci and gyri being significantly more similar between mono-zygotic twins than between control pairs. Higher order gyri and sulci show greater variability across individuals relative to lower order ones ^14,15,17^. Together, these data suggest that physical models may require additional influences to account for patterns of gyrification, and that genetic factors might regulate the emergence of cortical gyrification, to a certain degree in a deterministic fashion, especially of the primary, deeper sulci ^13,49,63^. In the next subsections, we will review the mechanical forces and cellular processes underlying cortical folding and discuss how the interactions between these mechanisms form the bases for the current model of cortical folding ^22,49,55,56,64,65^. This model proposes a central role for RGC dynamics in producing cortical gyration, based on a wealth of studies identifying genetic programs expressed in these progenitors during development that guide the expanding cortex to fold in a controlled manner. These developmental programs produce the necessary conditions for mechanical forces to fold the cortex—such as regions of relative cell production and expansion neighboring regions of slower growth—while maintaining minimal stochasticity for the development of primary gyri; the increased variability in higher order gyri appears to reflect a greater degree of stochasticity in their formation. Studying the interplay between both factors (mechanical forces and genetic and cellular programs) is essential to gain a better understanding of the mechanisms that orchestrate folding in the developing cortex.

### Mechanical forces underlying cortical folding

Prior to 2010, numerous models of the physics of cortical folding were debated, including one in which external force from the skull caused folding of the developing cortex ^14^, or one in which internal tension forces from axonal growth pulled cortical regions into a wrinkled shape ^66,67^. However, these two hypotheses are not strongly supported by more recent data: the developing skull does not place mechanical constraint because cranial sutures do not ossify until the completion of brain growth, and reducing cranial pressure does not influence gyrification in animal models ^14^. However, influence in the reverse direction is possible, and pressure from an expanding brain or fluid compartment can increase head size, as seen in cases of macrocephaly, both in mice and humans ^14,68,69^. The axon tension model is also inconsistent with experimental investigations demonstrating that localized severing of axons in gyrencephalic brains during development does not influence cortical folding ^70^.

The prevailing physical model of cortical gyration is based on differential tangential growth of the basal portion of the developing cortex (CP and MZ) relative to the underlying germinal zones (VZ and SVZ) ^62^. The foundations for exploring this model were laid by observations across animals that cortical folding is best correlated with cortical grey matter thickness and surface area rather than with brain size ^71^, and computational models showing the sufficiency of differential tangential expansion to produce convolutions resembling gyri in structures like the developing cortex ^72,73^. Building on these analyses, studies with physical models using polymer gels constructed of an inner region that expands less than an outer basal region produced patterns of gyration that are grossly similar in scale and pattern to those seen in the human cortex ^74,75^. By using simple polymer gel expansion models with initial parameters derived from fetal MRI imaging at 20 weeks of gestation, these physical models yielded striking similarity to human cortical gyration patterns ^76^. Variations in the stiffness of the inner and outer regions of these compound gel structures impacted the pattern and wavelength of folding, suggesting that the material properties of the tissue play key roles in folding ^75,76^. These findings are consistent with the notion that mechanical force from differential tangential expansion of the outer layer (CP and MZ) relative to the inner layer (VZ and SVZ) of the developing cortex is sufficient to cause folding in a pattern similar to that of the human cortex.

Even though studies of the physical causes of cortical gyration are compelling, they do not capture all the features of cortical folding seen *in vivo*. First, while mechanical models of cortical folding can correctly predict how folds arise from specific initial conditions, presumably these initial conditions explain the recurrence of specific folding patterns across individuals; and hence still require mechanistic understanding. Second, mechanical models do not yet encompass the physical properties of the distinct developmental layers and only recently have started to incorporate contributions that influence folding at microscopic rather than macroscopic levels, although it is striking how accurate they are already.. Finally, these models require tuning of initial parameters such as specific thickness of the layers modeled and rate of expansion pulled from biological estimates, and changes in these parameters have dramatic impacts on the outcomes of predicted gyrification ^76,77^.

### Roles of radial glial cells in cortical folding

**Figure 2.**
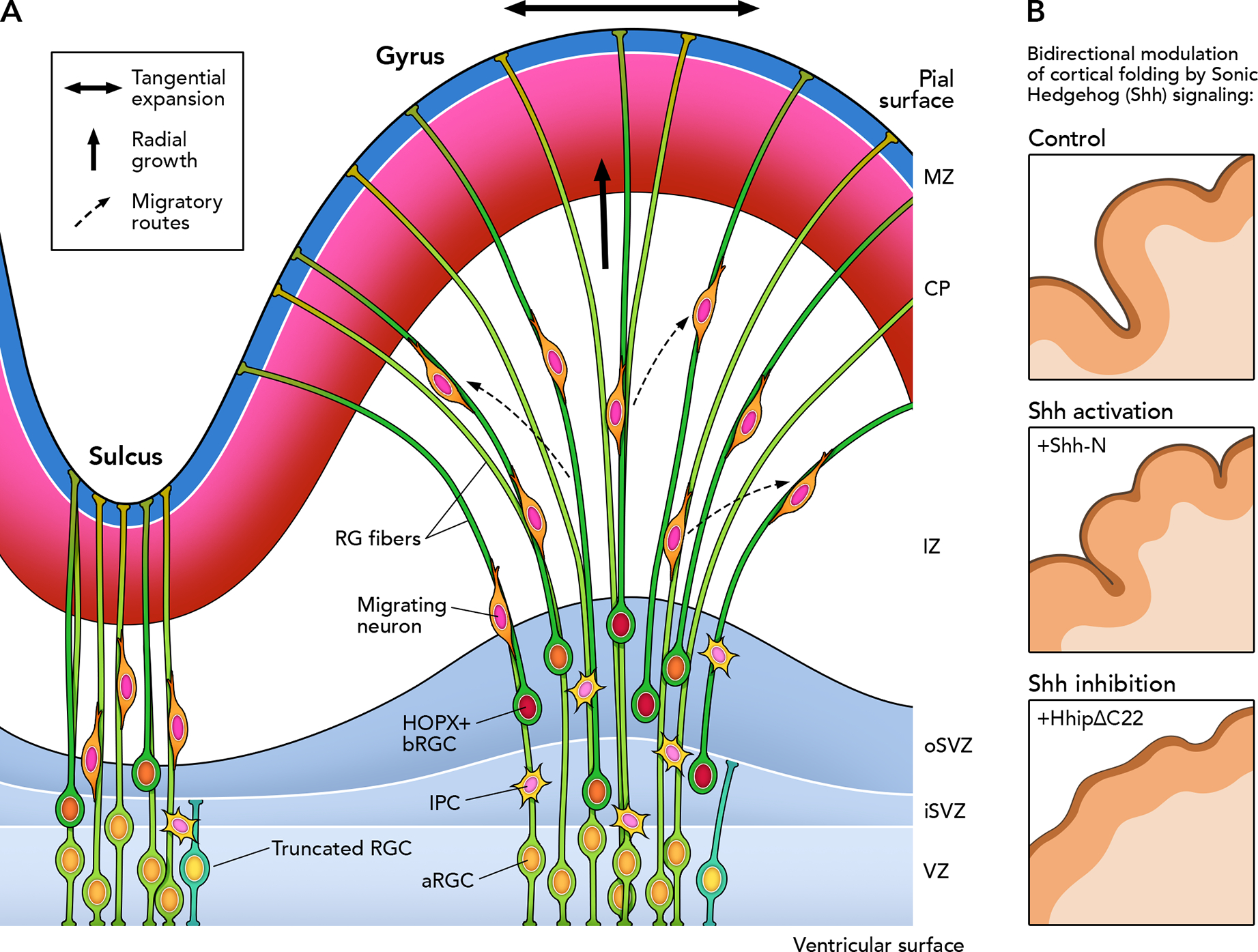
Cellular and molecular mechanisms of radial glial cells promote cortical folding. (**A**) The current model of cortical folding proposes a key role for basal radial glial cells (bRGCs), which are characterized by a highly proliferative capacity and are especially abundant in the outer subventricular zone (oSVZ). Regional differences in neurogenesis are found across the developing cortical mantle in gyrencephalic species (especially noticeable in the oSVZ): regions destined to prospective gyrus (proto-gyri) have greater densities of diving progenitor cells than regions destined to prospective sulci (proto-sulci) ^40,80^. Consistently, a subclass of bRGCs labeled by the marker HOPX in the developing ferret cortex is found in greater densities in proto-gyri ^31^. The differential density of basal progenitors results in differential production and accumulation of neurons in the developing cortical plate (CP), which causes a greater degree of tangential expansion (horizontal double arrow) in proto-gyri relative to proto-sulci. Moreover, there is a progressive divergence in the trajectory of radial glial (RG) fibers during gyrus formation due to the highly proliferative population of bRGCs that intercalate their fibers with preexisting ones ^40^. This fanned array of RG fibers in prospective gyral regions contributes to the tangential spread of migrating neurons guided by this scaffolding. In contrast, radial glial fibers typically show parallel trajectories in proto-sulci, which likely limits the tangential spread of migrating neurons. (**B**) Schematic illustrating the effects of Sonic Hedgehog (Shh) signaling in cortical folding. HOPX+ bRGCs were shown to respond to activation and inhibition of Shh signaling activity in inverse ways: Shh stimulation (with the amino-terminal fragment of Shh, Shh-N) results in increased densities of HOPX+ bRGCs, whereas Shh suppression (with the competitive inhibitor HhipΔ22) results in a reduced population of HOPX+ bRGCs ^31^. These effects lead to bidirectional changes in cortical folding: Shh stimulation results in larger gyri, whereas Shh inhibition leads to smaller gyri ^31^.

If differential tangential expansion during cortical development exerts mechanical forces that can induce folding, what is the cellular substrate responsible for the emergence of such forces in the developing cortex? Regional variation in neuronal density is viewed as a determinant factor in the differential growth of the cortical mantle ^49,56^. Not only can this differential accumulation of neurons can lead to region-specific rates of tangential expansion of the cortical surface area, but also the subsequent maturation of neurons (e.g., dendritic arbor development) has been suggested to further contribute to differential growth in developing gyri and sulci ^78^. Multiple lines of evidence over the past decade support a major role for the behavior of cortical progenitors (proliferation and neurogenesis) in the differential accumulation of neurons, ultimately influencing cortical folding. Initial anatomical analyses of primate developing cortices showed regional differences in the size of germinal layers, in particular the oSVZ ^45^. Subsequent studies demonstrated that the distribution of dividing cells is heterogeneous across the cortical mantle in gyrencephalic species, observations consistently reported in ferrets and monkeys ^40,79,80^, and several studies documented the differential distribution of these basal progenitors in regions that will generate prospective gyri (proto-gyri) and regions destined to become prospective sulci (proto-sulci) ^39,40,81^ (Figure 2A). The cellular density in the oSVZ relative to other germinal layers during development predicts the overall degree of cortical folding across mammalian species, independently of cortical size, such that the size of the oSVZ in development is not just correlated with brain size but also specifically with the gyrification index ^40,41^. More recently, experimental manipulations showed that increasing or reducing proliferation of bRGCs in ferrets leads to bidirectional modulation of cortical folding ^40,81–83^, and amplifying the limited pool of bRGCs in mice results in some degree of cortical folding ^84–86^ (further discussed in the following sections).

These findings formed the bases for a proposed mechanism underlying the emergence of primary gyri and primary sulci: elevated rates of neurogenesis in proto-gyri during corticogenesis will result in a higher accumulation of neurons and rapid tangential expansion of the CP, whereas lower rates of neurogenesis in proto-sulci (neighboring regions to proto-gyri) will lead to a lesser degree of accumulation of neurons and tangential growth, thus the developing cortex will begin to form folds and fissures (Figure 2A). A potential source for differential bRGC distribution across the developing cortex, and perhaps even local bRGC behavior, may be the differential gene expression observed between proto-gyri and proto-sulci in the developing cortex, and such expression patterns (also called protomaps of gene expression) are proposed to reflect the presence of early region-specific genetic programs guiding cortical folding and arealization in a stereotypical manner ^13,63,87,88^. It remains to be investigated whether the development of tertiary or higher order gyri—characteristic of highly folded cortices such as the human cortex and evidently variable across individuals—follow similar principles than the formation of primary gyri, which are present in ferrets, the animal model used in most experimental investigations into gyrification mechanisms so far.

Neurogenesis from bRGCs, which explains part of the broad mechanism of differential tangential expansion, does not appear to account for the degree of gyrification seen *in vivo* entirely ^62^. Aside from proliferation, bRGCs have also been found to contribute to cortical folding through the RG fiber scaffold they provide to nascent neurons, which guides and defines their radial migration and final positioning in the CP. Once bRGCs are initially born from aRGCs by delamination from the ventricular surface (roughly at embryonic day 34 in ferrets, and gestational week 12 in humans), their basal RG fibers begin to intercalate with those from preexisting aRGCs ^39–41^. The progressive accumulation of basal processes adds complexity to the fiber arrangement, causing fanning outward of the RG scaffold (Figure 2A). This is more prominent in regions with increased bRGC abundance, which correlates with prospective gyri ^39,40^. These data suggest that bRGCs contribute to differential tangential expansion of the CP not only by increasing the neurogenic potential, but also by promoting tangential dispersion of neurons in the developing cortex by increasing the complexity of the RG fiber network. Additionally, aRGCs appear to transform their basal processes at gestational week 16.5 in humans to a shorter fiber that becomes truncated at the border with the oSVZ, referred to as “truncated” RGCs, with these truncated progenitors presumably producing many late-born neurons ^35^. These truncated RGCs define a RG scaffold in humans that becomes discontinuous or interrupted between the iSVZ and oSVZ (with the short process of truncated aRGCs spanning the depth of the VZ and iSVZ and the long process of bRGCs extending from the oSVZ up to the CP) in the second trimester of gestation, posing intriguing questions about the migration and dispersion of late-born neurons and how this phenomenon may shape cortical architecture.

In addition to these RGC-driven mechanisms, there is evidence that mechanisms intrinsic to migrating neurons can also influence cortical folding. Migrating neurons associate with numerous fibers of the RG scaffold promiscuously to take more tortuous and divergent paths (by branching their leading process) in developing gyrencephalic cortices than they do in developing lissencephalic cortices, where clonal analyses of migrating neurons show a more limited conical distribution ^40,51,52,89,90^. Accordingly, the expression of FLRT1 and FLRT3—genes encoding cell adhesion molecules—by migrating neurons in mice appears to restrict their tangential dispersion and their deletion leads to the emergence of cortical sulci; in contrast, the downregulated expression of FLRT1/3 found in ferrets and humans was suggested to be a regulatory mechanism potentially underlying the distinct migration patterns observed in the gyrencephalic neocortex ^91,92^. Thus, the architecture of the RG fiber scaffold, together with cell-autonomous mechanisms of neuronal migration, critically influences cortical folding by impacting the tangential dispersion of migrating neurons.

## MOLECULAR MEDIATORS REGULATING RADIAL GLIAL CELL BEHAVIOR

Experimental studies in both mice and ferrets have provided direct evidence of the role of RGC behavior, and especially of bRGCs, in cortical folding and identified molecular signaling pathways involved in the genetic regulation of RGC function. Several ectopically expressed factors in mouse cortex can induce pseudogyration (folding of the entire developing cortical thickness including ventricular surface, usually due to increased size), but not convincing true ectopic gyration (cortical folding without disruption of the apical membrane shape, usually due to increased tangential dispersion). Thus, parallel experiments in ferrets investigating those same factors have now examined them in an animal model—the ferret—with the cellular substrate to modulate true cortical gyration. For example, local overexpression of fibroblast growth factor 2 (FGF2) or FGF8 via *in utero* electroporation in mice is sufficient to cause local growth reminiscent of gyration, attributed to increased self-renewal of aRGCs, the inverse of the phenotype described in an *Fgf2* knock-out (KO) mouse model ^93,94^. A similar experiment of FGF8 misexpression in ferrets caused frank polymicrogyria (a cortical malformation characterized by many small gyri creating excessive folding), and histological examination revealed an increased density of bRGCs not seen in the mouse experiments that is potentially driving the change in gyration ^95^. In a similar pattern, modulating cell cycle of RGCs through overexpression of the cell cycle proteins Cdk4/CyclinD1 via *in utero* electroporation in mice promoted increased *Tbr2*+ IPCs in the SVZ, which increases cortical surface area in mice, but did not distinctly lead to local gyri-reminiscent growth ^96^. Remarkably, the same manipulation in ferrets generates new cortical folds with normal laminar organization ^81^. These differences in phenotypes between mouse and ferret models may be explained by the distinct cytoarchitecture of the gyrencephalic developing cortex, characterized by expanded basal progenitors in the SVZ, that might be necessary for such phenotype. Alternatively, the underlying molecular programs involved in this phenotype in ferrets may not be shared by the mouse developing cortex ^81,96^. A unique case of a single gene perturbation in mice causing a phenotype of true cortical gyration is the loss of Trnp1 in the embryonic mouse cortex, loss of which led to an expansion of the small population of bRGC-like progenitors found in mice, thus causing an expanded oSVZ, increased fanning of the local RG scaffolding, and formation of cortical folds ^84^. Importantly, the cortical folding induced in these mice emerged from radial growth of the germinal zone followed by the tangential expansion of neurons in the cortical plate, recapitulating the endogenous process of gyrification described in the gyrencephalic cortex ^84^.

Beyond these examples of single gene perturbations in both murine and gyrencephalic models, numerous other genetic factors have been shown to modulate RGC behavior through studies using *in utero* electroporation, KO models, or drug treatments [excellently reviewed by ^65^]. These factors, including *IGF1* ^97^, *INSM1* ^98^, NT3 ^94^, lysophosphatidic acid ^99^, or modulation of beta-catenin signaling ^68^, modulate RGC proliferation, and can cause cortical lamination or even cortical shape changes, although none of these induce true gyration in their murine models. Thus far it seems that modulating genetic factors in lissencephalic animal models lacking the key cellular components of the developing cortex required for folding (that is, an abundant population of bRGCs) may not be sufficient to induce true cortical gyration. Likely, given the evolutionary history of mice and the emergence of pseudogyration in most experiments described above, it seems most plausible that mice have shed or suppressed much of the cellular substrate needed to form folds during corticogenesis and, as a result, developed a lissencephalic cortex. Thus, future experiments in gyrencephalic models such as ferret may be able to examine gyrification mechanisms specifically in a developing cortex with more endogenous substrate required for cortical folding.

Molecular cues from the extracellular environment have also been found to play a crucial role in cortical progenitor behavior, hence influencing cortical shape. In this context, the constituents of the extracellular matrix (ECM) are essential for cellular and tissue organization and dynamics in the human developing cortex, and in particular the proliferation of cortical progenitors, which has been thoroughly reviewed elsewhere ^100,101^. These functions of ECM proteins have been shown to be mediated both by modulating the activity of major signaling pathways (e.g., FGF signaling and Hedgehog signaling) and by direct signaling via ECM receptors (e.g., laminin binding to integrins). A seminal study identified three ECM components (HAPLN1, luminican and collagen I) involved in regulating the morphogenesis of the human developing cortex: treatment of 11–16 gestation week fetal neocortical slices with these ECM proteins as soluble factors induced folding within 24h of culture ^102^. Importantly, signaling mediated by integrins is required to promote the proliferation of both types of basal progenitors (bRGCs and bIPCs) in ferrets and mice ^44,103–105^. Beyond ECM-related molecular signaling, other cell-extrinsic factors, such as retinoic acid, have been shown to regulate proliferation of RGCs ^106^. Taken together, extracellular signals are essential in modulating the behavior of cortical progenitors and, therefore, act as determinant factors in the emergence of shape in the developing cerebral cortex.

## SONIC HEDGEHOG SIGNALING AND CORTICAL GYRATION

Recent findings indicate that Sonic hedgehog (Shh) signaling is a key regulator of cortical gyration through its function in bRGCs, further extending the broad spectrum of Shh roles in nervous system development (such as its well described role in the specification of the dorsal-ventral axis of the neural tube and spinal cord as well as cortical patterning). Shh is the best studied of the three hedgehog signaling ligands and its expression is expanded in its breadth and concentration in the developing cortices of gyrencephalic animals relative to lissencephalic ones ^31,107^. Shh acts as a mitogen in the developing cortex and promotes the proliferation of RGCs, stimulating their amplification over neurogenic divisions ^108–110^. While Shh might act as a mitogen in aRGCs in some capacity early in neurogenesis, its effects are more apparent on the proliferative capacity and stemness of bRGCs and IPCs in the SVZ and on the generation of bRGCs from aRGCs ^42,47,111^. Shh function in the SVZ promotes the capacity of bRGCs and IPCs to self-renew through symmetric divisions rather than terminally differentiate, thereby expanding bRGC and IPC populations ^31^. The role of Shh as a mitogen can be illustrated further by case examples that examined the effects of titrating Shh signaling activity during cortical development: (1) increased Shh signaling by constitutively activating the pathway in RGCs caused macrocephaly and even some cortical folding (pseudogyration) in mice ^86^; (2) decreased Shh due to defective Shh secretion into the cerebrospinal fluid led to a smaller neocortex and cerebellar hypoplasia due to decreased RGC proliferation in *CHMP1A*-null mice ^112^; and (3) prenatal treatment of ferrets with Shh signaling modulators in the critical window for bRGC production fine-tuned the density and dynamics of bRGCs: an Shh inhibitor led to decreased bRGC abundance, and an Shh agonist resulted in augmented bRGC pool size ^111^. This experiment did not induce noticeable effects on the aRGC population, suggesting a specific role for Shh signaling activity in basal progenitors ^111^.

Recent work has described greater radial glial diversity in gyrencephalic mammals (ferret, macaque, and human) relative to lissencephalic mice ^40,42,59^. The specific sensitivity to Shh stimulation by a subclass of bRGCs expressing *HOPX* (encoding a homeodomain-containing protein) has been recently reported. The HOPX+ bRGC subclass has greatly expanded in gyrencephalic mammals and accumulate in proto-gyri in higher numbers compared to the HOPX− bRGC subclass ^31^ (Figure 2A). Importantly, Shh activity specifically suppressed differentiation of HOPX+ bRGCs, thus increasing their neurogenic potential through progenitor self-renewal ^31^. Local suppression of Shh signaling in ferrets using a competitive inhibitor of Shh (HhipΔC22) led to decreased HOPX+ bRGCs and locally decreased cortical folding, demonstrating a necessary and sufficient relationship between Shh signaling, local gyrification, and the sustained local proliferation of bRGCs ^31^ (Figure 2B). Overall, in agreement with cortical folding theory described above, these studies show the potential of HOPX+ bRGCs to promote local differential tangential expansion through an Shh-mediated mechanism inducing elevated proliferation in proto-gyri. These results extend pioneer studies showing that ectopic constitutive Shh stimulation in the VZ in mice was sufficient to form cortical folds in the otherwise lissencephalic brain ^86^. Altogether, the function of Shh signaling pathway in HOPX+ bRGCs emerges as a central molecular mediator of cortical folding.

Evidence for the key role of Shh signaling in cortical folding also comes from described cortical malformation syndromes in which cortical gyration is abnormal. Although mutations in *SHH* have been traditionally associated with forebrain patterning malformations such as holoprosencephaly (a disorder in which the embryonic forebrain fails to form two distinct hemispheres), they have also been associated with microcephaly, polymicrogyria (characterized aberrant number of small folds), and schizencephaly (characterized by abnormal slits or clefts), as part of the phenotypic spectrum associated with dysfunctional Shh signaling ^113–115^. Mutations in genes associated with the Shh signaling pathway, such as receptors *PTCH1* and *CDON*, have also been associated with cortical malformations ^115–117^.

In addition, mutations in genes involved in modulating Shh signaling have also been linked to non-holoprosencephaly cortical malformations, providing further support to the notion that disrupted Shh signaling can lead to defects in cortical folding. These include the microcephaly/cerebellar hypoplasia associated with recessive mutations in *CHMP1A* ^112,118^, and the recently described polymicrogyria syndrome caused by bi-allelic mutations in *TMEM161B* ^119,120^. While disruption of *TMEM161B* through bi-allelic missense mutations in humans causes cortical gyration abnormalities, loss of *Tmem161b* function in mice causes several Shh-related malformations such as holoprosencephaly, eye defects, and craniofacial abnormalities, as well as morphologically abnormal primary cilia in the ventricular zone of the developing cortex ^119^. During cortical development, *TMEM161B* is expressed preferentially in HOPX+ bRGCs relative to other bRGC populations. Disrupting Tmem161b function in cortical progenitors in ferrets led to decreased gyral size and sulci depth, confirming its role in promoting normal cortical gyration ^119^. Furthermore, Tmem161b plays also a role in the integrity of the RG fiber scaffold, which as described above is different locally between proto-gyri and proto-sulci ^120^. Overall, these findings, especially in context of the ferret studies examining Shh stimulation of HOPX+ bRGCs described above, suggest a decreased sensitivity to Shh signaling in a developing gyrencephalic cortex deficient in Tmem161b, which provides important insights into the pathogenic mechanisms underlying the polymicrogyria syndrome described in patients.

More generally than the example of polymicrogyria associated with *TMEM161B* mutation, cortical malformations associated with dysfunction in the primary cilium (the key cellular organelle necessary for downstream transcriptional activation induced by Shh ligand), frequently include microcephaly and polymicrogyria as part of their phenotypic spectra ^121,122^. Although primary cilia also are involved in numerous other signaling processes, disruption of normal Shh signaling due to abnormal primary cilia may be in part explanatory for the cortical folding malformations seen in primary ciliopathies such as Joubert or Meckel-Gruber Syndrome ^121,122^.

## EVOLUTIONARY EXPANSION OF RADIAL GLIAL CELLS

**Figure 3.**
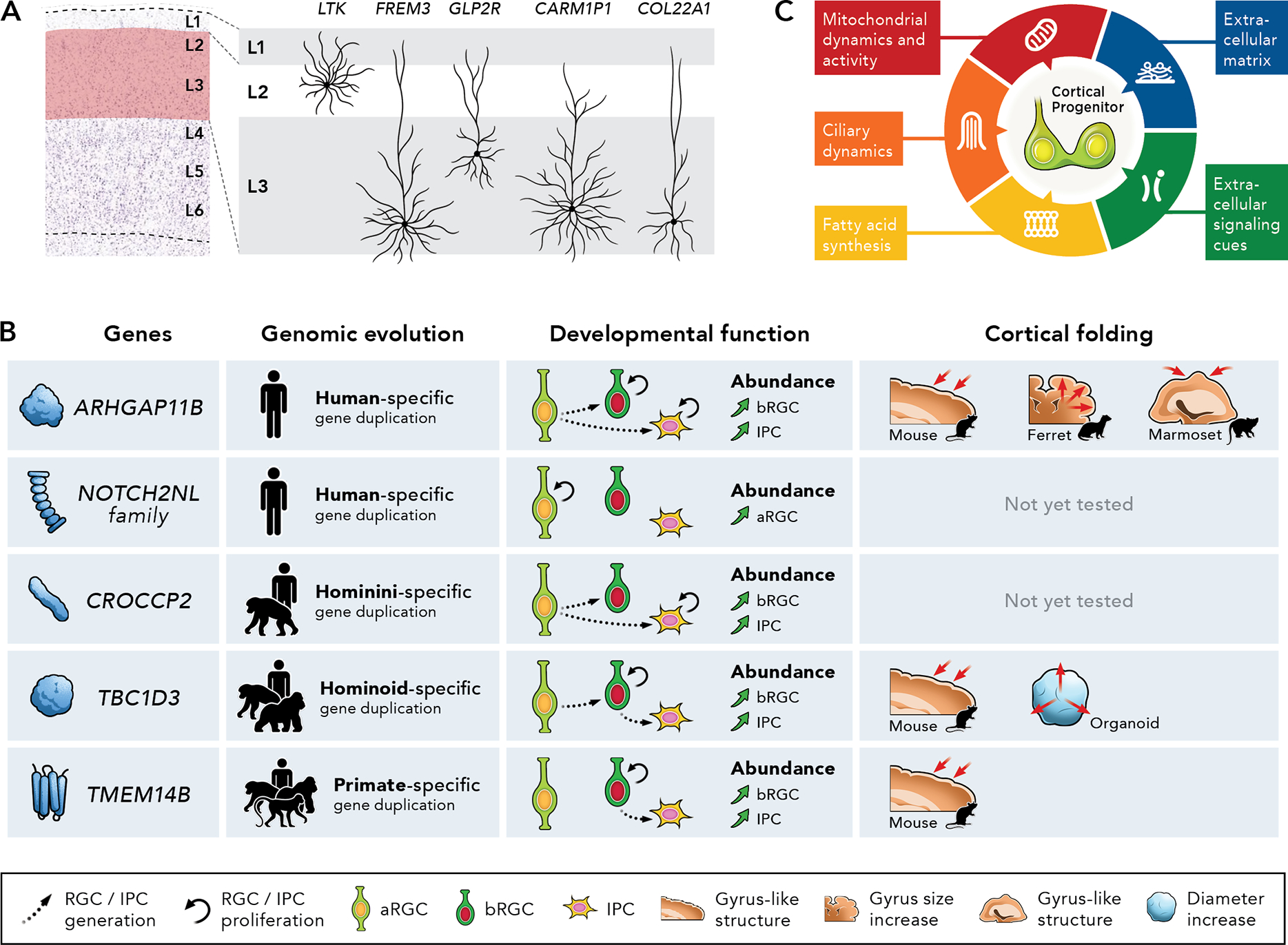
Evolutionary expansion of the human neocortex and cortical progenitors. (**A**) A hallmark of human neocortical evolution is the expansion of superficial layer neurons. Left, image of a coronal section of the human neocortex illustrating the relative thickness of cortical layers (L1-L6). Red shade highlights the expansion of superficial layers, especially L2–3, in the human neocortex. Image adapted from Allen Institute for Brain Science. Image credit: Allen Institute for Brain Science: https://human.brain-map.org/ish/specimen/show/79946224 (specifically, Nissl-stained brain section from the right-hand side panel: Image 7 of 8). Right, drawing illustrating the expanded neuronal diversity found in superficial layers of the human neocortex [as described in ^3^]; labels indicate the marker genes of specific types of excitatory pyramidal cells. (**B**) Gene duplications in evolution that regulate the behavior of cortical progenitors. *ARHGAP11B* arose 5 million years ago by partial duplication of *ARHGAP11A*. Three human-specific paralogs (*NOTCH2NLA*, *NOTCH2NLB*, and *NOTCH2NLC*) derived from duplication of *NOTCH2*. *CROCCP2* arose through partial duplication of exons 13–21 of *CROCC* in the Hominini lineage (chimpanzees and humans). *TBC1D3* arose in the Hominoid lineage (great apes) from a segmental duplication, with multiple copies found in the human genome and only a single copy found in the chimpanzee genome. *TMEM14B* arose in the primate lineage, including Old World and New World monkeys and apes. In parallel, experimental manipulations in mice, ferrets, and marmosets, as well as cerebral cortical organoids, have revealed a role for these genes in inducing cortical folding induction and/or cortical size expansion. Symbols represent the animal models in which cortical folding appeared as a result of overexpression experiments. (**C**) Distinctive features of cortical progenitors during human brain development. The schema compiles 5 cellular features reviewed in this work: extracellular matrix components, responsiveness to extracellular signaling cues, regulation of fatty acid synthesis, mitochondrial dynamics and metabolism, and ciliary dynamics and trafficking.

The evolution of neocortical circuits is thought to have enabled remarkable computational abilities required for human higher cognitive function ^123,124^. These evolutionary changes include the expansion of the neocortical cytoarchitecture (i.e., the cellular composition and distribution), and such enlargement is especially prominent in superficial layers 2 and 3 (also known as supragranular layers given their location above the granular layer 4) ^125^ (Figure 3A). Indeed, comparative transcriptomic analyses recently showed an extraordinary diversification of projection neuronal types within superficial layers of the human neocortex ^3^ (Figure 3A). The enlargement and diversification of superficial layer neurons may, therefore, provide a biological substrate for increased intracortical, associative connectivity that is essential in higher cognitive abilities, given that superficial layer neurons form cortico-cortical synaptic connections. As discussed above, numerous studies have documented that bRGCs are more numerous and diverse in gyrencephalic species as compared to lissencephalic species and, in particular, the oSVZ is substantially expanded in the primate developing cortex ^39,40,42–45^. Therefore, it is thought that the expansion of basal progenitors might underlie changes in neocortical cytoarchitecture that occurred over the course of evolution. The last decade has seen a quest for human-specific genomic changes that shaped the development of the cerebral cortex by controlling the behavior of cortical progenitors, thus elucidating molecular mechanisms that played an important role in human cortical evolution.

Gene duplication is a major evolutionary driving force. Many recent studies have documented the essential function that the emergence of novel genes during evolution played in the specialized behavior of human basal progenitors (thoroughly reviewed in ^56,126^) (Figure 3B). *ARGHAP11B*, a gene that arose on the human lineage after the divergence from the chimpanzee lineage by partial duplication of the Rho guanosine triphosphatase-activating protein *ARHGAP11A* and subsequently underwent a splice-site mutation, increases basal progenitor proliferation, as revealed by overexpression experiments in mice, ferrets, and marmosets ^82,85,127^. *CROCCP2*, a gene duplicated in the hominin lineage (chimpanzees and humans) that is highly expressed during human corticogenesis, enhances the generation of basal progenitors ^128^. Three human-specific paralogs derived from duplication of *NOTCH2* (*NOTCH2NLA*, *NOTCH2NLB*, and *NOTCH2NLC*) play critical roles in expanding the pool of cortical progenitors ^129,130^. The overexpression of *TBC1D3* (a great ape-specific gene) and of *TMEM14B* (a primate-specific gene) in mice promote the expansion of basal progenitors ^131–133^. As a result of the effects in basal progenitors, an increasing body of research in ferret models has revealed roles for many of these human-evolved genes in promoting both cortical neuron production and cortical folding (Figure 3).

Changes in gene regulation constitute another major force driving cortical evolution. A broad range of regulatory mechanisms have been found to be impacted not only by species-specific genomic changes during evolution but also by genetic variants associated with neurodevelopmental disorders. These range from mutations in enhancers and promoters to changes in alternative splicing ^134–138^, from the emergence of novel microRNAs and to that of novel long non-coding RNAs ^139–141^. Gene expression changes in the developing cortex can occur at different spatiotemporal scales, including cell types, cortical areas, and developmental windows ^59,88,142–145^. Such fine-tuned expression patterns likely reflect a positive selection necessary for precise functional organization of the cerebral cortex. Importantly, experimental manipulation in mice has shown a direct link between gene regulation and cortical expansion: the human accelerated region HARE5 upregulates the expression of FZD8, a receptor of the Wnt signaling pathway, and promotes accelerated cell cycle of cortical progenitors resulting in increased cortical size ^137^. Despite the increasing number of evolutionarily dynamic non-coding regions linked to cortical development, the functional evaluation of these loci in animal models remains limited and, therefore, their impact in regulating cortical shape and folding is still poorly understood.

Notably, several recent studies have revealed specialized molecular and cellular features of human cortical progenitors (Figure 3C). These include elevated expression of extracellular matrix proteins ^44,102,146^, responsiveness to extracellular signaling cues through innovations in receptor expression ^147–149^, molecular pathways controlling fatty acid synthesis ^83^, specialized mitochondrial dynamics and metabolism ^150,151^, and ciliary dynamics and trafficking ^128^. These mechanisms have been shown to modulate a variety of developmental processes, including not only the behavior of cortical progenitors but also the cell fate during neurogenesis as well as the protracted timing of neuronal maturation. Therefore, it is foreseeable that research over the next years will continue to decode evolutionary genetic mechanisms underlying these developmental processes, thus shedding new light into the organization of the human cerebral cortex. The recent development of novel comparative genomic strategies will provide new and exciting opportunities to approach these questions ^152,153^.

## CONCLUSIONS AND PERSPECTIVES

In the past decade, a wave of studies have revealed genetic mechanisms that—as discussed in this Review—regulate the highly proliferative capacity of basal radial glial cells and their effect on differential tangential expansion of the developing cortex, thereby establishing a direct link between the behavior of cortical progenitors to the emergence of cerebral cortical folding. Notwithstanding their significance, the study of how the human cerebral cortex acquires its shape during development appears to be at its beginning rather than its end. Advances in comparative genomics have identified a rapidly increasing number of candidate human-specific regulators of cortical development, both in coding and non-coding regions of the genome ^130,138,154^. Neuroimaging approaches coupled with genome-wide analyses have uncovered many genomic loci significantly associated with various neuroimaging phenotypes related to morphological variables of sulci and gyri ^155^. Single-cell transcriptomic analyses have found striking differences in gene expression across cortical regions and developmental stages, revealing molecular and cellular patterns for cortical arealization ^88,142–144^. Furthermore, it is striking to note the many variations in cortical gyrification patterns across mammalian species ^156–158^, the evolutionary specializations of defined cortical areas (especially within the frontal lobe) ^159–162^, as well as the effects of environmental perturbations in cortical cytoarchitecture ^40,163^. Altogether, these data suggest that only a minor fraction of genetic programs involved in shaping the structural and functional organization of the neocortex has been functionally dissected so far, as recently discussed in the context of the evolution of cortical connectivity ^124^, and many questions remained to be addressed. How do region-specific programs give rise to distinct functional cortical areas? What temporal windows define the specification of these areas? Which evolutionary mechanisms determine species-specific variation in cortical shape and organization? We think that the next years will see intense research on the mechanisms of the emergence of structural and functional organization in the neocortex, and that these developmental insights will have deep implications for our understanding of human cognitive functions.

Recent work is beginning to shed new light on additional cellular processes involved in shaping neocortical architecture. In later stages of development, cortical progenitors acquire new roles and shift their neurogenic fate into a gliogenic one, becoming committed to generate astrocytes as well as oligodendrocytes ^34^. A recent study found an important role for localized astrogenesis in gyri formation, and this process is regulated by FGF signaling ^164^. This is consistent with earlier observations in the macaque developing cortex that suggested a contribution of gliogenesis in folding of the cortical surface ^165^. Since glial cells represent extraordinarily voluminous populations in the neocortex ^7,8^, further understanding of the role of gliogenesis in cortical folding will complement the current knowledge on neurogenesis-driven mechanisms and lead to more comprehensive models on the emergence of shape during cortical development.

Finally, technological and bioinformatic advances, together with increased sample sizes, have spurred the identification of genetic variants associated with cortical malformations, implicating rapidly increasing numbers of genetic factors in recent years ^166^. This emphasizes the need to functionally interrogate novel genetic variants to continue dissecting the pathogenesis of these neurodevelopmental disorders. These studies will also be critical in expanding our understanding of molecular and cellular mechanisms involved in the development of cortical shape.

## References

[1] Sousa AMM, Meyer KA, Santpere G, Gulden FO, and Sestan N (2017). Evolution of the Human Nervous System Function, Structure, and Development. Cell 170, 226–247. 28708995 10.1016/j.cell.2017.06.036 PMC5647789

[2] Kaas J (2020). Evolutionary Neuroscience 2nd ed. Kaas J, ed. (Academic Press).

[3] Berg J, Sorensen SA, Ting JT, Miller JA, Chartrand T, Buchin A, Bakken TE, Budzillo A, Dee N, Ding S-L, (2021). Human neocortical expansion involves glutamatergic neuron diversification. Nature 598, 151–158. 34616067 10.1038/s41586-021-03813-8 PMC8494638

[4] Bakken TE, Jorstad NL, Hu Q, Lake BB, Tian W, Kalmbach BE, Crow M, Hodge RD, Krienen FM, Sorensen SA, (2021). Comparative cellular analysis of motor cortex in human, marmoset and mouse. Nature 598, 111–119. 34616062 10.1038/s41586-021-03465-8 PMC8494640

[5] Jorstad NL, Song JHT, Exposito-Alonso D, Suresh H, Castro N, Krienen FM, Yanny AM, Close J, Gelfand E, Travaglini KJ, (2022). Comparative transcriptomics reveals human-specific cortical features. bioRxiv, 2022.09.19.508480. 10.1101/2022.09.19.508480. PMC10659116 37824638

[6] Ma S, Skarica M, Li Q, Xu C, Risgaard RD, Tebbenkamp ATN, Mato-Blanco X, Kovner R, Krsnik Ž, de Martin X, (2022). Molecular and cellular evolution of the primate dorsolateral prefrontal cortex. Science, eabo7257. 36007006 10.1126/science.abo7257 PMC9614553

[7] Herculano-Houzel S (2009). The human brain in numbers: a linearly scaled-up primate brain. Front. Hum. Neurosci. 3, 31. 19915731 10.3389/neuro.09.031.2009 PMC2776484

[8] Sherwood CC, Stimpson CD, Raghanti MA, Wildman DE, Uddin M, Grossman LI, Goodman M, Redmond JC, Bonar CJ, Erwin JM, (2006). Evolution of increased glia-neuron ratios in the human frontal cortex. Proc. Natl. Acad. Sci. U. S. A. 103, 13606–13611. 16938869 10.1073/pnas.0605843103 PMC1564260

[9] Jayaraman D, Bae B-I, and Walsh CA (2018). The Genetics of Primary Microcephaly. Annu. Rev. Genomics Hum. Genet. 19, 177–200. 29799801 10.1146/annurev-genom-083117-021441

[10] Faheem M, Naseer MI, Rasool M, Chaudhary AG, Kumosani TA, Ilyas AM, Pushparaj P, Ahmed F, Algahtani HA, Al-Qahtani MH, (2015). Molecular genetics of human primary microcephaly: an overview. BMC Med. Genomics 8 Suppl 1, S4. 10.1186/1755-8794-8-S1-S4 PMC4315316 25951892

[11] Tanaka T, and Gleeson JG (2000). Genetics of brain development and malformation syndromes. Curr. Opin. Pediatr. 12, 523–528. 11106269 10.1097/00008480-200012000-00002

[12] Hu WF, Chahrour MH, and Walsh CA (2014). The diverse genetic landscape of neurodevelopmental disorders. Annu. Rev. Genomics Hum. Genet. 15, 195–213. 25184530 10.1146/annurev-genom-090413-025600 PMC10591257

[13] de Juan Romero C, and Borrell V (2017). Genetic maps and patterns of cerebral cortex folding. Curr. Opin. Cell Biol. 49, 31–37. 29227862 10.1016/j.ceb.2017.11.009

[14] Welker W (1990). Why Does Cerebral Cortex Fissure and Fold? In Cerebral Cortex: Comparative Structure and Evolution of Cerebral Cortex, Part II, Jones EG and Peters A, eds. (Springer US), pp. 3–136.

[15] Lohmann G, von Cramon DY, and Steinmetz H (1999). Sulcal variability of twins. Cereb. Cortex 9, 754–763. 10554998 10.1093/cercor/9.7.754

[16] Lohmann G, von Cramon DY, and Colchester ACF (2008). Deep sulcal landmarks provide an organizing framework for human cortical folding. Cereb. Cortex 18, 1415–1420. 17921455 10.1093/cercor/bhm174

[17] Schmitt JE, Raznahan A, Liu S, and Neale MC (2021). The Heritability of Cortical Folding: Evidence from the Human Connectome Project. Cereb. Cortex 31, 702–715. 32959043 10.1093/cercor/bhaa254 PMC7727360

[18] Miller JA, D’Esposito M, and Weiner KS (2021). Using tertiary sulci to map the “cognitive globe” of prefrontal cortex. J. Cogn. Neurosci. 33, 1698–1715. 34375416 10.1162/jocn_a_01696

[19] Cachia A, Borst G, Jardri R, Raznahan A, Murray GK, Mangin J-F, and Plaze M (2021). Towards Deciphering the Fetal Foundation of Normal Cognition and Cognitive Symptoms From Sulcation of the Cortex. Front. Neuroanat. 15, 712862. 34650408 10.3389/fnana.2021.712862 PMC8505772

[20] Hilgetag CC, and Barbas H (2006). Role of mechanical factors in the morphology of the primate cerebral cortex. PLoS Comput. Biol. 2, e22. 16557292 10.1371/journal.pcbi.0020022 PMC1409812

[21] Klyachko VA, and Stevens CF (2003). Connectivity optimization and the positioning of cortical areas. Proc. Natl. Acad. Sci. U. S. A. 100, 7937–7941. 12796510 10.1073/pnas.0932745100 PMC164691

[22] Zilles K, Palomero-Gallagher N, and Amunts K (2013). Development of cortical folding during evolution and ontogeny. Trends Neurosci. 36, 275–284. 23415112 10.1016/j.tins.2013.01.006

[23] Ashwell KWS, and Hardman CD (2012). Distinct development of the cerebral cortex in platypus and echidna. Brain Behav. Evol. 79, 57–72. 22143038 10.1159/000334188

[24] Dehay C, Kennedy H, and Kosik KS (2015). The outer subventricular zone and primate-specific cortical complexification. Neuron 85, 683–694. 25695268 10.1016/j.neuron.2014.12.060

[25] Lewitus E, Kelava I, Kalinka AT, Tomancak P, and Huttner WB (2014). An adaptive threshold in mammalian neocortical evolution. PLoS Biol. 12, e1002000. 25405475 10.1371/journal.pbio.1002000 PMC4236020

[26] O’Leary MA, Bloch JI, Flynn JJ, Gaudin TJ, Giallombardo A, Giannini NP, Goldberg SL, Kraatz BP, Luo Z-X, Meng J, (2013). The placental mammal ancestor and the post-K-Pg radiation of placentals. Science 339, 662–667. 23393258 10.1126/science.1229237

[27] Smart IH, and McSherry GM (1986). Gyrus formation in the cerebral cortex in the ferret. I. Description of the external changes. J. Anat. 146, 141–152. 3693054 PMC1166530

[28] Akula SK, Chen AY, (2023). Exome sequencing and the identification of new genes and shared mechanisms in polymicrogyria. JAMA Neurol. *(*accepted*)*. 10.1001/jamaneurol.2023.2363 PMC10366952 37486637

[29] Gilardi C, and Kalebic N (2021). The Ferret as a Model System for Neocortex Development and Evolution. Front Cell Dev Biol 9, 661759. 33996819 10.3389/fcell.2021.661759 PMC8118648

[30] Johnson MB, Sun X, Kodani A, Borges-Monroy R, Girskis KM, Ryu SC, Wang PP, Patel K, Gonzalez DM, Woo YM, (2018). Aspm knockout ferret reveals an evolutionary mechanism governing cerebral cortical size. Nature 556, 370–375. 29643508 10.1038/s41586-018-0035-0 PMC6095461

[31] Matsumoto N, Tanaka S, Horiike T, Shinmyo Y, and Kawasaki H (2020). A discrete subtype of neural progenitor crucial for cortical folding in the gyrencephalic mammalian brain. Elife 9. 10.7554/eLife.54873. PMC7173966 32312384

[32] Noctor SC, Martínez-Cerdeño V, Ivic L, and Kriegstein AR (2004). Cortical neurons arise in symmetric and asymmetric division zones and migrate through specific phases. Nat. Neurosci. 7, 136–144. 14703572 10.1038/nn1172

[33] Noctor SC, Martínez-Cerdeño V, and Kriegstein AR (2008). Distinct behaviors of neural stem and progenitor cells underlie cortical neurogenesis. J. Comp. Neurol. 508, 28–44. 18288691 10.1002/cne.21669 PMC2635107

[34] Kriegstein A, and Alvarez-Buylla A (2009). The glial nature of embryonic and adult neural stem cells. Annu. Rev. Neurosci. 32, 149–184. 19555289 10.1146/annurev.neuro.051508.135600 PMC3086722

[35] Nowakowski TJ, Pollen AA, Sandoval-Espinosa C, and Kriegstein AR (2016). Transformation of the Radial Glia Scaffold Demarcates Two Stages of Human Cerebral Cortex Development. Neuron 91, 1219–1227. 27657449 10.1016/j.neuron.2016.09.005 PMC5087333

[36] Haubensak W, Attardo A, Denk W, and Huttner WB (2004). Neurons arise in the basal neuroepithelium of the early mammalian telencephalon: a major site of neurogenesis. Proc. Natl. Acad. Sci. U. S. A. 101, 3196–3201. 14963232 10.1073/pnas.0308600100 PMC365766

[37] Miyata T, Kawaguchi A, Saito K, Kawano M, Muto T, and Ogawa M (2004). Asymmetric production of surface-dividing and non-surface-dividing cortical progenitor cells. Development 131, 3133–3145. 15175243 10.1242/dev.01173

[38] Pebworth M-P, Ross J, Andrews M, Bhaduri A, and Kriegstein AR (2021). Human intermediate progenitor diversity during cortical development. Proc. Natl. Acad. Sci. U. S. A. 118. 10.1073/pnas.2019415118. PMC8256045 34155100

[39] Reillo I, and Borrell V (2012). Germinal zones in the developing cerebral cortex of ferret: ontogeny, cell cycle kinetics, and diversity of progenitors. Cereb. Cortex 22, 2039–2054. 21988826 10.1093/cercor/bhr284

[40] Reillo I, de Juan Romero C, García-Cabezas MÁ, and Borrell V (2011). A role for intermediate radial glia in the tangential expansion of the mammalian cerebral cortex. Cereb. Cortex 21, 1674–1694. 21127018 10.1093/cercor/bhq238

[41] Pilz G-A, Shitamukai A, Reillo I, Pacary E, Schwausch J, Stahl R, Ninkovic J, Snippert HJ, Clevers H, Godinho L, (2013). Amplification of progenitors in the mammalian telencephalon includes a new radial glial cell type. Nat. Commun. 4, 2125. 23839311 10.1038/ncomms3125 PMC3717501

[42] Betizeau M, Cortay V, Patti D, Pfister S, Gautier E, Bellemin-Ménard A, Afanassieff M, Huissoud C, Douglas RJ, Kennedy H, (2013). Precursor diversity and complexity of lineage relationships in the outer subventricular zone of the primate. Neuron 80, 442–457. 24139044 10.1016/j.neuron.2013.09.032

[43] Hansen DV, Lui JH, Parker PRL, and Kriegstein AR (2010). Neurogenic radial glia in the outer subventricular zone of human neocortex. Nature 464, 554–561. 20154730 10.1038/nature08845

[44] Fietz SA, Kelava I, Vogt J, Wilsch-Bräuninger M, Stenzel D, Fish JL, Corbeil D, Riehn A, Distler W, Nitsch R, (2010). OSVZ progenitors of human and ferret neocortex are epithelial-like and expand by integrin signaling. Nat. Neurosci. 13, 690–699. 20436478 10.1038/nn.2553

[45] Smart IHM, Dehay C, Giroud P, Berland M, and Kennedy H (2002). Unique morphological features of the proliferative zones and postmitotic compartments of the neural epithelium giving rise to striate and extrastriate cortex in the monkey. Cereb. Cortex 12, 37–53. 11734531 10.1093/cercor/12.1.37 PMC1931430

[46] LaMonica BE, Lui JH, Hansen DV, and Kriegstein AR (2013). Mitotic spindle orientation predicts outer radial glial cell generation in human neocortex. Nat. Commun. 4, 1665. 23575669 10.1038/ncomms2647 PMC3625970

[47] Shitamukai A, Konno D, and Matsuzaki F (2011). Oblique radial glial divisions in the developing mouse neocortex induce self-renewing progenitors outside the germinal zone that resemble primate outer subventricular zone progenitors. J. Neurosci. 31, 3683–3695. 21389223 10.1523/JNEUROSCI.4773-10.2011 PMC6622781

[48] Fietz SA, and Huttner WB (2011). Cortical progenitor expansion, self-renewal and neurogenesis-a polarized perspective. Curr. Opin. Neurobiol. 21, 23–35. 21036598 10.1016/j.conb.2010.10.002

[49] Llinares-Benadero C, and Borrell V (2019). Deconstructing cortical folding: genetic, cellular and mechanical determinants. Nat. Rev. Neurosci. 20, 161–176. 30610227 10.1038/s41583-018-0112-2

[50] Rakic P (1988). Specification of cerebral cortical areas. Science 241, 170–176. 3291116 10.1126/science.3291116

[51] Gertz CC, and Kriegstein AR (2015). Neuronal Migration Dynamics in the Developing Ferret Cortex. J. Neurosci. 35, 14307–14315. 26490868 10.1523/JNEUROSCI.2198-15.2015 PMC4683689

[52] Ware ML, Tavazoie SF, Reid CB, and Walsh CA (1999). Coexistence of widespread clones and large radial clones in early embryonic ferret cortex. Cereb. Cortex 9, 636–645. 10498282 10.1093/cercor/9.6.636

[53] Kornack DR, and Rakic P (1995). Radial and horizontal deployment of clonally related cells in the primate neocortex: relationship to distinct mitotic lineages. Neuron 15, 311–321. 7646888 10.1016/0896-6273(95)90036-5

[54] Noctor SC, Flint AC, Weissman TA, Dammerman RS, and Kriegstein AR (2001). Neurons derived from radial glial cells establish radial units in neocortex. Nature 409, 714–720. 11217860 10.1038/35055553

[55] Taverna E, Götz M, and Huttner WB (2014). The cell biology of neurogenesis: toward an understanding of the development and evolution of the neocortex. Annu. Rev. Cell Dev. Biol. 30, 465–502. 25000993 10.1146/annurev-cellbio-101011-155801

[56] Pinson A, and Huttner WB (2021). Neocortex expansion in development and evolution-from genes to progenitor cell biology. Curr. Opin. Cell Biol. 73, 9–18. 34098196 10.1016/j.ceb.2021.04.008

[57] Cárdenas A, and Borrell V (2020). Molecular and cellular evolution of corticogenesis in amniotes. Cell. Mol. Life Sci. 77, 1435–1460. 31563997 10.1007/s00018-019-03315-x PMC11104948

[58] Namba T, and Huttner WB (2017). Neural progenitor cells and their role in the development and evolutionary expansion of the neocortex. Wiley Interdiscip. Rev. Dev. Biol. 6. 10.1002/wdev.256. 27865053

[59] Pollen AA, Nowakowski TJ, Chen J, Retallack H, Sandoval-Espinosa C, Nicholas CR, Shuga J, Liu SJ, Oldham MC, Diaz A, (2015). Molecular identity of human outer radial glia during cortical development. Cell 163, 55–67. 26406371 10.1016/j.cell.2015.09.004 PMC4583716

[60] Wang X, Tsai J-W, LaMonica B, and Kriegstein AR (2011). A new subtype of progenitor cell in the mouse embryonic neocortex. Nat. Neurosci. 14, 555–561. 21478886 10.1038/nn.2807 PMC3083489

[61] Stepien BK, Vaid S, and Huttner WB (2021). Length of the Neurogenic Period-A Key Determinant for the Generation of Upper-Layer Neurons During Neocortex Development and Evolution. Front Cell Dev Biol 9, 676911. 34055808 10.3389/fcell.2021.676911 PMC8155536

[62] Kroenke CD, and Bayly PV (2018). How Forces Fold the Cerebral Cortex. J. Neurosci. 38, 767–775. 29367287 10.1523/JNEUROSCI.1105-17.2017 PMC5783962

[63] de Juan Romero C, Bruder C, Tomasello U, Sanz-Anquela JM, and Borrell V (2015). Discrete domains of gene expression in germinal layers distinguish the development of gyrencephaly. EMBO J. 34, 1859–1874. 25916825 10.15252/embj.201591176 PMC4547892

[64] Lui JH, Hansen DV, and Kriegstein AR (2011). Development and evolution of the human neocortex. Cell 146, 18–36. 21729779 10.1016/j.cell.2011.06.030 PMC3610574

[65] Del-Valle-Anton L, and Borrell V (2022). Folding brains: from development to disease modeling. Physiol. Rev. 102, 511–550. 34632805 10.1152/physrev.00016.2021

[66] Bray D (1984). Axonal growth in response to experimentally applied mechanical tension. Dev. Biol. 102, 379–389. 6706005 10.1016/0012-1606(84)90202-1

[67] Van Essen DC (1997). A tension-based theory of morphogenesis and compact wiring in the central nervous system. Nature 385, 313–318. 9002514 10.1038/385313a0

[68] Chenn A, and Walsh CA (2002). Regulation of cerebral cortical size by control of cell cycle exit in neural precursors. Science 297, 365–369. 12130776 10.1126/science.1074192

[69] Jones SG, and Samanta D (2022). Macrocephaly (StatPearls Publishing). 32809621

[70] Xu G, Knutsen AK, Dikranian K, Kroenke CD, Bayly PV, and Taber LA (2010). Axons pull on the brain, but tension does not drive cortical folding. J. Biomech. Eng. 132, 071013. 20590291 10.1115/1.4001683 PMC3170872

[71] Mota B, and Herculano-Houzel S (2015). Cortical folding scales universally with surface area and thickness, not number of neurons. Science 349, 74–77. 26138976 10.1126/science.aaa9101

[72] Dervaux J, and Ben Amar M (2008). Morphogenesis of growing soft tissues. Phys. Rev. Lett. 101, 068101. 18764507 10.1103/PhysRevLett.101.068101

[73] Toro R, and Burnod Y (2005). A morphogenetic model for the development of cortical convolutions. Cereb. Cortex 15, 1900–1913. 15758198 10.1093/cercor/bhi068

[74] Bayly PV, Okamoto RJ, Xu G, Shi Y, and Taber LA (2013). A cortical folding model incorporating stress-dependent growth explains gyral wavelengths and stress patterns in the developing brain. Phys. Biol. 10, 016005. 23357794 10.1088/1478-3975/10/1/016005 PMC3616769

[75] Tallinen T, Chung JY, Biggins JS, and Mahadevan L (2014). Gyrification from constrained cortical expansion. Proc. Natl. Acad. Sci. U. S. A. 111, 12667–12672. 25136099 10.1073/pnas.1406015111 PMC4156754

[76] Tallinen T, Chung JY, Rousseau F, Girard N, Lefèvre J, and Mahadevan L (2016). On the growth and form of cortical convolutions. Nat. Phys. 12, 588–593.

[77] Budday S, Steinmann P, and Kuhl E (2014). The role of mechanics during brain development. J. Mech. Phys. Solids 72, 75–92. 25202162 10.1016/j.jmps.2014.07.010 PMC4156279

[78] Wang X, Studholme C, Grigsby PL, Frias AE, Cuzon Carlson VC, and Kroenke CD (2017). Folding, But Not Surface Area Expansion, Is Associated with Cellular Morphological Maturation in the Fetal Cerebral Cortex. J. Neurosci. 37, 1971–1983. 28069920 10.1523/JNEUROSCI.3157-16.2017 PMC5338750

[79] Lukaszewicz A, Savatier P, Cortay V, Giroud P, Huissoud C, Berland M, Kennedy H, and Dehay C (2005). G1 phase regulation, area-specific cell cycle control, and cytoarchitectonics in the primate cortex. Neuron 47, 353–364. 16055060 10.1016/j.neuron.2005.06.032 PMC1890568

[80] Lukaszewicz A, Cortay V, Giroud P, Berland M, Smart I, Kennedy H, and Dehay C (2006). The concerted modulation of proliferation and migration contributes to the specification of the cytoarchitecture and dimensions of cortical areas. Cereb. Cortex 16 Suppl 1, i26–34. 16766704 10.1093/cercor/bhk011

[81] Nonaka-Kinoshita M, Reillo I, Artegiani B, Martínez-Martínez MÁ, Nelson M, Borrell V, and Calegari F (2013). Regulation of cerebral cortex size and folding by expansion of basal progenitors. EMBO J. 32, 1817–1828. 23624932 10.1038/emboj.2013.96 PMC3926188

[82] Kalebic N, Gilardi C, Albert M, Namba T, Long KR, Kostic M, Langen B, and Huttner WB (2018). Human-specific ARHGAP11B induces hallmarks of neocortical expansion in developing ferret neocortex. Elife 7. 10.7554/eLife.41241. PMC6303107 30484771

[83] Pinson A, Xing L, Namba T, Kalebic N, Peters J, Oegema CE, Traikov S, Reppe K, Riesenberg S, Maricic T, (2022). Human TKTL1 implies greater neurogenesis in frontal neocortex of modern humans than Neanderthals. Science 377, eabl6422. 36074851 10.1126/science.abl6422

[84] Stahl R, Walcher T, De Juan Romero C, Pilz GA, Cappello S, Irmler M, Sanz-Aquela JM, Beckers J, Blum R, Borrell V, (2013). Trnp1 regulates expansion and folding of the mammalian cerebral cortex by control of radial glial fate. Cell 153, 535–549. 23622239 10.1016/j.cell.2013.03.027

[85] Florio M, Albert M, Taverna E, Namba T, Brandl H, Lewitus E, Haffner C, Sykes A, Wong FK, Peters J, (2015). Human-specific gene ARHGAP11B promotes basal progenitor amplification and neocortex expansion. Science 347, 1465–1470. 25721503 10.1126/science.aaa1975

[86] Wang L, Hou S, and Han Y-G (2016). Hedgehog signaling promotes basal progenitor expansion and the growth and folding of the neocortex. Nat. Neurosci. 19, 888–896. 27214567 10.1038/nn.4307 PMC4925239

[87] Elsen GE, Hodge RD, Bedogni F, Daza RAM, Nelson BR, Shiba N, Reiner SL, and Hevner RF (2013). The protomap is propagated to cortical plate neurons through an *Eomes-*dependent intermediate map. Proc. Natl. Acad. Sci. U. S. A. 110, 4081–4086. 23431145 10.1073/pnas.1209076110 PMC3593833

[88] Bhaduri A, Sandoval-Espinosa C, Otero-Garcia M, Oh I, Yin R, Eze UC, Nowakowski TJ, and Kriegstein AR (2021). An atlas of cortical arealization identifies dynamic molecular signatures. Nature 598, 200–204. 34616070 10.1038/s41586-021-03910-8 PMC8494648

[89] Martínez-Martínez MÁ, Ciceri G, Espinós A, Fernández V, Marín O, and Borrell V (2019). Extensive branching of radially-migrating neurons in the mammalian cerebral cortex. J. Comp. Neurol. 527, 1558–1576. 30520050 10.1002/cne.24597

[90] Reid CB, Liang I, and Walsh C (1995). Systematic widespread clonal organization in cerebral cortex. Neuron 15, 299–310. 7646887 10.1016/0896-6273(95)90035-7

[91] del Toro D, Ruff T, Cederfjäll E, Villalba A, Seyit-Bremer G, Borrell V, and Klein R (2017). Regulation of Cerebral Cortex Folding by Controlling Neuronal Migration via FLRT Adhesion Molecules. Cell 169, 621–635.e16. 28475893 10.1016/j.cell.2017.04.012

[92] Del Toro D, Carrasquero-Ordaz MA, Chu A, Ruff T, Shahin M, Jackson VA, Chavent M, Berbeira-Santana M, Seyit-Bremer G, Brignani S, (2020). Structural Basis of Teneurin-Latrophilin Interaction in Repulsive Guidance of Migrating Neurons. Cell 180, 323–339.e19. 31928845 10.1016/j.cell.2019.12.014 PMC6978801

[93] Rash BG, Lim HD, Breunig JJ, and Vaccarino FM (2011). FGF signaling expands embryonic cortical surface area by regulating Notch-dependent neurogenesis. J. Neurosci. 31, 15604–15617. 22031906 10.1523/JNEUROSCI.4439-11.2011 PMC3235689

[94] Lukaszewicz A, Savatier P, Cortay V, Kennedy H, and Dehay C (2002). Contrasting effects of basic fibroblast growth factor and neurotrophin 3 on cell cycle kinetics of mouse cortical stem cells. J. Neurosci. 22, 6610–6622. 12151540 10.1523/JNEUROSCI.22-15-06610.2002 PMC2001296

[95] Masuda K, Toda T, Shinmyo Y, Ebisu H, Hoshiba Y, Wakimoto M, Ichikawa Y, and Kawasaki H (2015). Pathophysiological analyses of cortical malformation using gyrencephalic mammals. Sci. Rep. 5, 15370. 26482531 10.1038/srep15370 PMC4613358

[96] Lange C, Huttner WB, and Calegari F (2009). Cdk4/cyclinD1 overexpression in neural stem cells shortens G1, delays neurogenesis, and promotes the generation and expansion of basal progenitors. Cell Stem Cell 5, 320–331. 19733543 10.1016/j.stem.2009.05.026

[97] Mairet-Coello G, Tury A, and DiCicco-Bloom E (2009). Insulin-like growth factor-1 promotes G(1)/S cell cycle progression through bidirectional regulation of cyclins and cyclin-dependent kinase inhibitors via the phosphatidylinositol 3-kinase/Akt pathway in developing rat cerebral cortex. J. Neurosci. 29, 775–788. 19158303 10.1523/JNEUROSCI.1700-08.2009 PMC3256126

[98] Tavano S, Taverna E, Kalebic N, Haffner C, Namba T, Dahl A, Wilsch-Bräuninger M, Paridaen JTML, and Huttner WB (2018). Insm1 Induces Neural Progenitor Delamination in Developing Neocortex via Downregulation of the Adherens Junction Belt-Specific Protein Plekha7. Neuron 97, 1299–1314.e8. 29503187 10.1016/j.neuron.2018.01.052

[99] Kingsbury MA, Rehen SK, Contos JJA, Higgins CM, and Chun J (2003). Non-proliferative effects of lysophosphatidic acid enhance cortical growth and folding. Nat. Neurosci. 6, 1292–1299. 14625558 10.1038/nn1157

[100] Long KR, and Huttner WB (2019). How the extracellular matrix shapes neural development. Open Biol. 9, 180216. 30958121 10.1098/rsob.180216 PMC6367132

[101] Amin S, and Borrell V (2020). The Extracellular Matrix in the Evolution of Cortical Development and Folding. Front Cell Dev Biol 8, 604448. 33344456 10.3389/fcell.2020.604448 PMC7744631

[102] Long KR, Newland B, Florio M, Kalebic N, Langen B, Kolterer A, Wimberger P, and Huttner WB (2018). Extracellular Matrix Components HAPLN1, Lumican, and Collagen I Cause Hyaluronic Acid-Dependent Folding of the Developing Human Neocortex. Neuron. 10.1016/j.neuron.2018.07.013. 30078576

[103] Stenzel D, Wilsch-Bräuninger M, Wong FK, Heuer H, and Huttner WB (2014). Integrin αvβ3 and thyroid hormones promote expansion of progenitors in embryonic neocortex. Development 141, 795–806. 24496617 10.1242/dev.101907

[104] Kalebic N, Gilardi C, Stepien B, Wilsch-Bräuninger M, Long KR, Namba T, Florio M, Langen B, Lombardot B, Shevchenko A, (2019). Neocortical Expansion Due to Increased Proliferation of Basal Progenitors Is Linked to Changes in Their Morphology. Cell Stem Cell 24, 535–550.e9. 30905618 10.1016/j.stem.2019.02.017

[105] Güven A, Kalebic N, Long KR, Florio M, Vaid S, Brandl H, Stenzel D, and Huttner WB (2020). Extracellular matrix-inducing Sox9 promotes both basal progenitor proliferation and gliogenesis in developing neocortex. Elife 9. 10.7554/eLife.49808. PMC7105383 32191207

[106] Siegenthaler JA, Ashique AM, Zarbalis K, Patterson KP, Hecht JH, Kane MA, Folias AE, Choe Y, May SR, Kume T, (2009). Retinoic acid from the meninges regulates cortical neuron generation. Cell 139, 597–609. 19879845 10.1016/j.cell.2009.10.004 PMC2772834

[107] Yabut OR, and Pleasure SJ (2018). Sonic Hedgehog Signaling Rises to the Surface: Emerging Roles in Neocortical Development. Brain Plast 3, 119–128. 30151337 10.3233/BPL-180064 PMC6091060

[108] Komada M (2012). Sonic hedgehog signaling coordinates the proliferation and differentiation of neural stem/progenitor cells by regulating cell cycle kinetics during development of the neocortex. Congenit. Anom. 52, 72–77. 10.1111/j.1741-4520.2012.00368.x 22639991

[109] Komada M, Saitsu H, Kinboshi M, Miura T, Shiota K, and Ishibashi M (2008). Hedgehog signaling is involved in development of the neocortex. Development 135, 2717–2727. 18614579 10.1242/dev.015891

[110] Saade M, Gutiérrez-Vallejo I, Le Dréau G, Rabadán MA, Miguez DG, Buceta J, and Martí E (2013). Sonic hedgehog signaling switches the mode of division in the developing nervous system. Cell Rep. 4, 492–503. 23891002 10.1016/j.celrep.2013.06.038

[111] Hou S, Ho W-L, Wang L, Kuo B, Park JY, and Han Y-G (2021). Biphasic Roles of Hedgehog Signaling in the Production and Self-Renewal of Outer Radial Glia in the Ferret Cerebral Cortex. Cereb. Cortex 31, 4730–4741. 34002221 10.1093/cercor/bhab119 PMC8408449

[112] Coulter ME, Dorobantu CM, Lodewijk GA, Delalande F, Cianferani S, Ganesh VS, Smith RS, Lim ET, Xu CS, Pang S, (2018). The ESCRT-III Protein CHMP1A Mediates Secretion of Sonic Hedgehog on a Distinctive Subtype of Extracellular Vesicles. Cell Rep. 24, 973–986.e8. 30044992 10.1016/j.celrep.2018.06.100 PMC6178983

[113] Nanni L, Ming JE, Bocian M, Steinhaus K, Bianchi DW, Die-Smulders C, Giannotti A, Imaizumi K, Jones KL, Campo MD, (1999). The mutational spectrum of the sonic hedgehog gene in holoprosencephaly: SHH mutations cause a significant proportion of autosomal dominant holoprosencephaly. Hum. Mol. Genet. 8, 2479–2488. 10556296 10.1093/hmg/8.13.2479

[114] Schell-Apacik CC, Ertl-Wagner B, Panzel A, Klausener K, Rausch G, Muenke M, von Voss H, and Hehr U (2009). Maternally inherited heterozygous sequence change in the sonic hedgehog gene in a male patient with bilateral closed-lip schizencephaly and partial absence of the corpus callosum. Am. J. Med. Genet. A 149A, 1592–1594. 19533790 10.1002/ajmg.a.32940 PMC2774861

[115] Hehr U, Pineda-Alvarez DE, Uyanik G, Hu P, Zhou N, Hehr A, Schell-Apacik C, Altus C, Daumer-Haas C, Meiner A, (2010). Heterozygous mutations in SIX3 and SHH are associated with schizencephaly and further expand the clinical spectrum of holoprosencephaly. Hum. Genet. 127, 555–561. 20157829 10.1007/s00439-010-0797-4 PMC4101187

[116] Ming JE, Kaupas ME, Roessler E, Brunner HG, Golabi M, Tekin M, Stratton RF, Sujansky E, Bale SJ, and Muenke M (2002). Mutations in PATCHED-1, the receptor for SONIC HEDGEHOG, are associated with holoprosencephaly. Hum. Genet. 110, 297–301. 11941477 10.1007/s00439-002-0695-5

[117] Bae G-U, Domené S, Roessler E, Schachter K, Kang J-S, Muenke M, and Krauss RS (2011). Mutations in CDON, encoding a hedgehog receptor, result in holoprosencephaly and defective interactions with other hedgehog receptors. Am. J. Hum. Genet. 89, 231–240. 21802063 10.1016/j.ajhg.2011.07.001 PMC3155179

[118] Mochida GH, Ganesh VS, de Michelena MI, Dias H, Atabay KD, Kathrein KL, Huang H-T, Hill RS, Felie JM, Rakiec D, (2012). CHMP1A encodes an essential regulator of BMI1-INK4A in cerebellar development. Nat. Genet. 44, 1260–1264. 23023333 10.1038/ng.2425 PMC3567443

[119] Akula SK, Marciano JH, Lim Y, Exposito-Alonso D, Hylton NK, Hwang GH, Neil JE, Dominado N, Bunton-Stasyshyn RK, Song JHT, (2023). TMEM161B regulates cerebral cortical gyration, Sonic Hedgehog signaling, and ciliary structure in the developing central nervous system. Proc. Natl. Acad. Sci. U. S. A. 120, e2209964120. 36669111 10.1073/pnas.2209964120 PMC9942790

[120] Wang L, Heffner C, loi Vong K, Barrows C, Ha Y-J, Lee S, Lara-Gonzalez P, Jhamb I, Meer DVD, Loughnan R, (2023). TMEM161B modulates radial glial scaffolding in neocortical development. Proc. Natl. Acad. Sci. U. S. A. 120, e2209983120. 36669109 10.1073/pnas.2209983120 PMC9942823

[121] Reiter JF, and Leroux MR (2017). Genes and molecular pathways underpinning ciliopathies. Nat. Rev. Mol. Cell Biol. 18, 533–547. 28698599 10.1038/nrm.2017.60 PMC5851292

[122] Andreu-Cervera A, Catala M, and Schneider-Maunoury S (2021). Cilia, ciliopathies and hedgehog-related forebrain developmental disorders. Neurobiol. Dis. 150, 105236. 33383187 10.1016/j.nbd.2020.105236

[123] Galakhova AA, Hunt S, Wilbers R, Heyer DB, de Kock CPJ, Mansvelder HD, and Goriounova NA (2022). Evolution of cortical neurons supporting human cognition. Trends Cogn. Sci. 26, 909–922. 36117080 10.1016/j.tics.2022.08.012 PMC9561064

[124] Vanderhaeghen P, and Polleux F (2023). Developmental mechanisms underlying the evolution of human cortical circuits. Nat. Rev. Neurosci. 10.1038/s41583-023-00675-z. PMC10064077 36792753

[125] Hutsler JJ, Lee D-G, and Porter KK (2005). Comparative analysis of cortical layering and supragranular layer enlargement in rodent carnivore and primate species. Brain Res. 1052, 71–81. 16018988 10.1016/j.brainres.2005.06.015

[126] Mora-Bermúdez F, and Huttner WB (2022). What Are the Human-Specific Aspects of Neocortex Development? Front. Neurosci. 16, 878950. 35495057 10.3389/fnins.2022.878950 PMC9047014

[127] Heide M, Haffner C, Murayama A, Kurotaki Y, Shinohara H, Okano H, Sasaki E, and Huttner WB (2020). Human-specific *ARHGAP11B* increases size and folding of primate neocortex in the fetal marmoset. Science 369, 546–550. 32554627 10.1126/science.abb2401

[128] Van Heurck R, Bonnefont J, Wojno M, Suzuki IK, Velez-Bravo FD, Erkol E, Nguyen DT, Herpoel A, Bilheu A, Beckers S, (2022). CROCCP2 acts as a human-specific modifier of cilia dynamics and mTOR signaling to promote expansion of cortical progenitors. Neuron. 10.1016/j.neuron.2022.10.018. PMC9831670 36334595

[129] Fiddes IT, Lodewijk GA, Mooring M, Bosworth CM, Ewing AD, Mantalas GL, Novak AM, van den Bout A, Bishara A, Rosenkrantz JL, (2018). Human-Specific NOTCH2NL Genes Affect Notch Signaling and Cortical Neurogenesis. Cell 173, 1356–1369.e22. 29856954 10.1016/j.cell.2018.03.051 PMC5986104

[130] Suzuki IK, Gacquer D, Van Heurck R, Kumar D, Wojno M, Bilheu A, Herpoel A, Lambert N, Cheron J, Polleux F, (2018). Human-Specific NOTCH2NL Genes Expand Cortical Neurogenesis through Delta/Notch Regulation. Cell 173, 1370–1384.e16. 29856955 10.1016/j.cell.2018.03.067 PMC6092419

[131] Hou Q-Q, Xiao Q, Sun X-Y, Ju X-C, and Luo Z-G (2021). TBC1D3 promotes neural progenitor proliferation by suppressing the histone methyltransferase G9a. Sci Adv 7. 10.1126/sciadv.aba8053. PMC7810367 33523893

[132] Ju X-C, Hou Q-Q, Sheng A-L, Wu K-Y, Zhou Y, Jin Y, Wen T, Yang Z, Wang X, and Luo Z-G (2016). The hominoid-specific gene TBC1D3 promotes generation of basal neural progenitors and induces cortical folding in mice. Elife 5. 10.7554/eLife.18197. PMC5028191 27504805

[133] Liu J, Liu W, Yang L, Wu Q, Zhang H, Fang A, Li L, Xu X, Sun L, Zhang J, (2017). The Primate-Specific Gene TMEM14B Marks Outer Radial Glia Cells and Promotes Cortical Expansion and Folding. Cell Stem Cell 21, 635–649.e8. 29033352 10.1016/j.stem.2017.08.013

[134] McLean CY, Reno PL, Pollen AA, Bassan AI, Capellini TD, Guenther C, Indjeian VB, Lim X, Menke DB, Schaar BT, (2011). Human-specific loss of regulatory DNA and the evolution of human-specific traits. Nature 471, 216–219. 21390129 10.1038/nature09774 PMC3071156

[135] Bae B-I, Tietjen I, Atabay KD, Evrony GD, Johnson MB, Asare E, Wang PP, Murayama AY, Im K, Lisgo SN, (2014). Evolutionarily dynamic alternative splicing of GPR56 regulates regional cerebral cortical patterning. Science 343, 764–768. 24531968 10.1126/science.1244392 PMC4480613

[136] Reilly SK, Yin J, Ayoub AE, Emera D, Leng J, Cotney J, Sarro R, Rakic P, and Noonan JP (2015). Evolutionary genomics. Evolutionary changes in promoter and enhancer activity during human corticogenesis. Science 347, 1155–1159. 25745175 10.1126/science.1260943 PMC4426903

[137] Boyd JL, Skove SL, Rouanet JP, Pilaz L-J, Bepler T, Gordân R, Wray GA, and Silver DL (2015). Human-chimpanzee differences in a FZD8 enhancer alter cell-cycle dynamics in the developing neocortex. Curr. Biol. 25, 772–779. 25702574 10.1016/j.cub.2015.01.041 PMC4366288

[138] Doan RN, Bae B-I, Cubelos B, Chang C, Hossain AA, Al-Saad S, Mukaddes NM, Oner O, Al-Saffar M, Balkhy S, (2016). Mutations in Human Accelerated Regions Disrupt Cognition and Social Behavior. Cell 167, 341–354.e12. 27667684 10.1016/j.cell.2016.08.071 PMC5063026

[139] Arcila ML, Betizeau M, Cambronne XA, Guzman E, Doerflinger N, Bouhallier F, Zhou H, Wu B, Rani N, Bassett DS, (2014). Novel primate miRNAs coevolved with ancient target genes in germinal zone-specific expression patterns. Neuron 81, 1255–1262. 24583023 10.1016/j.neuron.2014.01.017 PMC4020629

[140] Rani N, Nowakowski TJ, Zhou H, Godshalk SE, Lisi V, Kriegstein AR, and Kosik KS (2016). A Primate lncRNA Mediates Notch Signaling during Neuronal Development by Sequestering miRNA. Neuron 90, 1174–1188. 27263970 10.1016/j.neuron.2016.05.005 PMC4911262

[141] Nowakowski TJ, Rani N, Golkaram M, Zhou HR, Alvarado B, Huch K, West JA, Leyrat A, Pollen AA, Kriegstein AR, (2018). Regulation of cell-type-specific transcriptomes by microRNA networks during human brain development. Nat. Neurosci. 21, 1784–1792. 30455455 10.1038/s41593-018-0265-3 PMC6312854

[142] Nowakowski TJ, Bhaduri A, Pollen AA, Alvarado B, Mostajo-Radji MA, Di Lullo E, Haeussler M, Sandoval-Espinosa C, Liu SJ, Velmeshev D, (2017). Spatiotemporal gene expression trajectories reveal developmental hierarchies of the human cortex. Science 358, 1318–1323. 29217575 10.1126/science.aap8809 PMC5991609

[143] Zhu Y, Sousa AMM, Gao T, Skarica M, Li M, Santpere G, Esteller-Cucala P, Juan D, Ferrández-Peral L, Gulden FO, (2018). Spatiotemporal transcriptomic divergence across human and macaque brain development. Science 362. 10.1126/science.aat8077. PMC6900982 30545855

[144] Li M, Santpere G, Imamura Kawasawa Y, Evgrafov OV, Gulden FO, Pochareddy S, Sunkin SM, Li Z, Shin Y, Zhu Y, (2018). Integrative functional genomic analysis of human brain development and neuropsychiatric risks. Science 362. 10.1126/science.aat7615. PMC6413317 30545854

[145] Eze UC, Bhaduri A, Haeussler M, Nowakowski TJ, and Kriegstein AR (2021). Single-cell atlas of early human brain development highlights heterogeneity of human neuroepithelial cells and early radial glia. Nat. Neurosci. 24, 584–594. 33723434 10.1038/s41593-020-00794-1 PMC8012207

[146] Fietz SA, Lachmann R, Brandl H, Kircher M, Samusik N, Schröder R, Lakshmanaperumal N, Henry I, Vogt J, Riehn A, (2012). Transcriptomes of germinal zones of human and mouse fetal neocortex suggest a role of extracellular matrix in progenitor self-renewal. Proc. Natl. Acad. Sci. U. S. A. 109, 11836–11841. 22753484 10.1073/pnas.1209647109 PMC3406833

[147] Lui JH, Nowakowski TJ, Pollen AA, Javaherian A, Kriegstein AR, and Oldham MC (2014). Radial glia require PDGFD-PDGFRβ signalling in human but not mouse neocortex. Nature 515, 264–268. 25391964 10.1038/nature13973 PMC4231536

[148] Mayer S, Chen J, Velmeshev D, Mayer A, Eze UC, Bhaduri A, Cunha CE, Jung D, Arjun A, Li E, (2019). Multimodal Single-Cell Analysis Reveals Physiological Maturation in the Developing Human Neocortex. Neuron 102, 143–158.e7. 30770253 10.1016/j.neuron.2019.01.027 PMC7648658

[149] Xing L, Kalebic N, Namba T, Vaid S, Wimberger P, and Huttner WB (2020). Serotonin Receptor 2A Activation Promotes Evolutionarily Relevant Basal Progenitor Proliferation in the Developing Neocortex. Neuron 108, 1113–1129.e6. 33080227 10.1016/j.neuron.2020.09.034

[150] Iwata R, Casimir P, and Vanderhaeghen P (2020). Mitochondrial dynamics in postmitotic cells regulate neurogenesis. Science 369, 858–862. 32792401 10.1126/science.aba9760

[151] Iwata R, Casimir P, Erkol E, Boubakar L, Planque M, Gallego López IM, Ditkowska M, Gaspariunaite V, Beckers S, Remans D, (2023). Mitochondria metabolism sets the species-specific tempo of neuronal development. Science, eabn4705. 36705539 10.1126/science.abn4705

[152] Song JHT, Grant RL, Behrens VC, Kučka M, Roberts Kingman GA, Soltys V, Chan YF, and Kingsley DM (2021). Genetic studies of human-chimpanzee divergence using stem cell fusions. Proc. Natl. Acad. Sci. U. S. A. 118. 10.1073/pnas.2117557118. PMC8713981 34921118

[153] Agoglia RM, Sun D, Birey F, Yoon S-J, Miura Y, Sabatini K, Pașca SP, and Fraser HB (2021). Primate cell fusion disentangles gene regulatory divergence in neurodevelopment. Nature 592, 421–427. 33731928 10.1038/s41586-021-03343-3 PMC8719633

[154] Mangan RJ, Alsina FC, Mosti F, Sotelo-Fonseca JE, Snellings DA, Au EH, Carvalho J, Sathyan L, Johnson GD, Reddy TE, (2022). Adaptive sequence divergence forged new neurodevelopmental enhancers in humans. Cell 185, 4587–4603.e23. 36423581 10.1016/j.cell.2022.10.016 PMC10013929

[155] van der Meer D, Kaufmann T, Shadrin AA, Makowski C, Frei O, Roelfs D, Monereo-Sánchez J, Linden DEJ, Rokicki J, Alnæs D, (2021). The genetic architecture of human cortical folding. Sci Adv 7, eabj9446. 34910505 10.1126/sciadv.abj9446 PMC8673767

[156] DeFelipe J (2011). The Evolution of the Brain, the Human Nature of Cortical Circuits, and Intellectual Creativity. Front. Neuroanat. 5. 10.3389/fnana.2011.00029. PMC3098448 21647212

[157] Manger PR, Prowse M, Haagensen M, and Hemingway J (2012). Quantitative analysis of neocortical gyrencephaly in African elephants (Loxodonta africana) and six species of cetaceans: comparison with other mammals. J. Comp. Neurol. 520, 2430–2439. 22237903 10.1002/cne.23046

[158] Pillay P, and Manger PR (2007). Order-specific quantitative patterns of cortical gyrification. Eur. J. Neurosci. 25, 2705–2712. 17459107 10.1111/j.1460-9568.2007.05524.x

[159] Schenker NM, Buxhoeveden DP, Blackmon WL, Amunts K, Zilles K, and Semendeferi K (2008). A comparative quantitative analysis of cytoarchitecture and minicolumnar organization in Broca’s area in humans and great apes. J. Comp. Neurol. 510, 117–128. 18612968 10.1002/cne.21792

[160] Semendeferi K, Armstrong E, Schleicher A, Zilles K, and Van Hoesen GW (2001). Prefrontal cortex in humans and apes: a comparative study of area 10. Am. J. Phys. Anthropol. 114, 224–241. 11241188 10.1002/1096-8644(200103)114:3<224::AID-AJPA1022>3.0.CO;2-I

[161] Bauernfeind AL, de Sousa AA, Avasthi T, Dobson SD, Raghanti MA, Lewandowski AH, Zilles K, Semendeferi K, Allman JM, Craig ADB, (2013). A volumetric comparison of the insular cortex and its subregions in primates. J. Hum. Evol. 64, 263–279. 23466178 10.1016/j.jhevol.2012.12.003 PMC3756831

[162] Preuss TM, and Wise SP (2022). Evolution of prefrontal cortex. Neuropsychopharmacology 47, 3–19. 34363014 10.1038/s41386-021-01076-5 PMC8617185

[163] Dehay C, Giroud P, Berland M, Killackey H, and Kennedy H (1996). Contribution of thalamic input to the specification of cytoarchitectonic cortical fields in the primate: effects of bilateral enucleation in the fetal monkey on the boundaries, dimensions, and gyrification of striate and extrastriate cortex. J. Comp. Neurol. 367, 70–89. 8867284 10.1002/(SICI)1096-9861(19960325)367:1<70::AID-CNE6>3.0.CO;2-G

[164] Shinmyo Y, Saito K, Hamabe-Horiike T, Kameya N, Ando A, Kawasaki K, Duong TAD, Sakashita M, Roboon J, Hattori T, (2022). Localized astrogenesis regulates gyrification of the cerebral cortex. Sci Adv 8, eabi5209. 35275722 10.1126/sciadv.abi5209 PMC8916738

[165] Rash BG, Duque A, Morozov YM, Arellano JI, Micali N, and Rakic P (2019). Gliogenesis in the outer subventricular zone promotes enlargement and gyrification of the primate cerebrum. Proc. Natl. Acad. Sci. U. S. A. 116, 7089–7094. 30894491 10.1073/pnas.1822169116 PMC6452694

[166] Chung C, Yang X, Bae T, Vong KI, Mittal S, Donkels C, Westley Phillips H, Li Z, Marsh APL, Breuss MW, (2023). Comprehensive multi-omic profiling of somatic mutations in malformations of cortical development. Nat. Genet, 1–12. 10.1038/s41588-022-01276-9 PMC9961399 36635388

